# A Fatigue-Life Correction Model for Finite Element Analysis of Flexible Composite Structures Under Fatigue Loading

**DOI:** 10.3390/ma19204365

**Published:** 2026-10-17

**Authors:** Jie Zhou, Qi Liu, Xiaowei Xu, Yubin Lu, Zhen Wu

**Affiliations:** 1 AVIC Xi’an Aircraft Industry Group Company Ltd., Xi’an 710089, China; 2 School of Aeronautics, Northwestern Polytechnical University, Xi’an 710072, China; 3 National Key Laboratory of Strength and Structural Integrity, Xi’an 710065, China

**Keywords:** progressive fatigue damage, finite element method, fatigue-life correction model, artificial neural network, glass-fiber-reinforced polymer (GFRP), fatigue loading

## Abstract

Fatigue-life prediction of flexible composite structures using finite element analysis can be affected by discrepancies between conventional fatigue-life models obtained from standard specimen tests and their application at the finite-element integration-point level. In this study, a fatigue-life correction model (FLCM) is proposed to reduce this discrepancy by considering the evolution of the fatigue state during cyclic loading. The FLCM incorporates damage-induced stiffness degradation, local stress redistribution, and the resulting evolution of the effective stress level into the fatigue-life formulation, thereby providing a correction to the conventional fatigue-life relationship when it is applied in finite element fatigue analysis. An artificial-neural-network-based fatigue stress–damage mapping method (FSDM-ANN) is further developed to represent the relationship between stress level, normalized fatigue life, and fatigue stiffness degradation, and is integrated with the FLCM to establish a fatigue progressive damage model for finite element analysis. The proposed approach is implemented in ABAQUS through a user-defined material subroutine and evaluated using flexible composite structures subjected to combined tensile–bending loading. The experimental program includes two static tests and two fatigue tests, with the fatigue tests performed using staged displacement amplitudes. The finite element predictions are compared with the experimental results in terms of structural load–cycle response and normalized fatigue stiffness degradation. The normalized fatigue stiffness error is 2.93% for the FLCM, compared with 7.04% for the conventional fatigue-life model. The results indicate that the proposed fatigue-life correction formulation can reduce the fatigue-life prediction discrepancy associated with the direct application of conventional fatigue-life data in finite element analysis and can support fatigue prediction under varying effective stress levels within the calibrated material and structural conditions.

## 1. Introduction

Fiber-reinforced polymers (FRPs) with high specific strength and stiffness are widely used in aircraft engineering. Aeronautical structures are commonly subjected to high-cycle fatigue loads during service [1,2]. In particular, laminated composite structures with complex geometry encounter more severe challenges of multiaxial fatigue damage [3]. Therefore, in order to accurately predict the process of fatigue progressive damage, it is very necessary to investigate the fatigue theory model, fatigue failure mechanism, and three-dimensional finite element model (3D-FEM).

Within the framework of fatigue theory models, many fatigue-life models (FLMs) [4,5,6,7] and fatigue progressive damage models (FPDMs) [8,9,10] for FRPs have been established. The typical 3D FLM was developed by Shokrieh and Lessard [11,12], which is universal for different laminated FRPs to evaluate fatigue life. Joo and Kim [13] established a fatigue-life evaluation technology depending on the mechanical contribution of fiber and matrix, which can accurately predict the fatigue life of unidirectional (UD) composites. Gao et al. [14] presented a pair of residual strength and residual stiffness models based on a unified fatigue damage formula with high accuracy. Naderi and Maligno [15] developed a residual stiffness and strength model to predict the fatigue life of carbon-fiber-reinforced polymers (CFRP), which are randomly generated by a Gaussian distribution function. Considering the disadvantages of imprecision, instability, and single applicability of traditional FPDMs, Hu et al. [16] developed a novel S–N curve model and a residual strength degradation model based on the statistical distribution.

Recently, machine learning algorithms (MLAs) have been widely applied to address the issues of FRP fatigue [17] and mechanical performance [18,19], in which the artificial neural network (ANN) [20] is a typical algorithm. Fortunately, the special properties of the fatigue data exactly satisfy the requirements of ANN models (ANNMs). Lee et al. [21] established ANNMs to predict the fatigue life of CFRP and GFRP laminates under constant loading and block loading conditions, which provides a prototype model for other investigators. Furthermore, Kadi and Al-Assaf [22,23] have also made significant contributions to the fatigue-life prediction of UD GFRP laminates using ANN. Kumar and Swamy [24] applied an ANN method to illustrate the effect of stress levels on fatigue strength and failure modes of GFRP. Marani and Nehdi [25] proposed a novel framework to predict the shear capacity of FRP-reinforced concrete beams using MLAs.

Moreover, investigators have attempted to clarify the fatigue failure mechanism. In terms of failure modes, Ganesan and Joanna [26] proposed a two-parameter model to predict the residual strength and fatigue life in tension–tension (T–T) fatigue for CFRPs. Sarker et al. [27] evaluated the fatigue life and fracture behaviors of CFRP laminates under compression–compression (C–C) loading conditions. In addition, Koloor et al. [28] introduced a cyclic cohesive zone model (CCZM) to predict fatigue damage of interfaces for FRPs. Lei et al. [29] extended the Iosipescu method to analyze the shear fatigue properties and reveal the process of shear failure for CFRPs. Sharma and Singh [30] studied the fatigue behavior of symmetric and asymmetric GFRP laminates. In terms of loading spectrum, Ma et al. [31] developed a single-parameter model to predict the variation tendency and distribution regularity of fatigue experimental data points under different stress ratios and loading frequencies. Brod et al. [32] proposed a fatigue damage model for UD FRPs relying on an energy-based method, which has a good capacity for predicting fatigue life under block loading conditions with arbitrary load sequences. Zheng et al. [33] developed an adaptive cyclic jump algorithm to balance the accuracy of life predictions and computational efficiency.

In order to establish FPDMs for complex working conditions and structures, Maneval et al. [34] proposed an FPDM based on the Palmgren–Miner accumulation rule to describe the fatigue failure of open-hole laminates under tensile cyclic load. Choi et al. [35] presented an ANN model to predict the split growth of notched laminates under constant-amplitude and spectrum fatigue, which performs better than the traditional power-law model. In addition, Zhou et al. [36] developed an FPDM by combining fatigue master curves with cohesive fatigue failure criteria. The FEM based on the proposed model reproduced fatigue behaviors of notched GFRP and CFRP composite bolted joints. Lian et al. [37] established a novel multiaxial fatigue-life prediction model based on the matrix shear failure mechanism under multiaxial fatigue loading, which was verified by UD GFRP tubular specimens under combined tension–torsion loading.

By reviewing the existing literature, it can be seen that substantial efforts have been devoted to fatigue-life prediction, progressive fatigue damage modeling, residual stiffness and strength degradation, and effective-stress-based descriptions of fatigue behavior in fiber-reinforced polymer composites. These approaches provide important tools for describing fatigue damage accumulation and the degradation of material properties during cyclic loading. However, a specific issue remains when fatigue-life relations obtained from standard specimen tests are directly introduced into finite element fatigue analysis. In conventional fatigue tests, the fatigue life *N_f_* is established under a prescribed nominal stress level, whereas in a progressive finite element analysis the stress state at an individual integration point can evolve as fatigue damage develops. In this study, four stress concepts are distinguished to clarify this issue. Nominal applied stress refers to the externally prescribed stress associated with the applied structural load. Local element stress refers to the stress calculated at a specific finite-element integration point and may vary spatially and evolve during damage accumulation. Stress level *S* denotes the normalized stress parameter used to establish the conventional fatigue-life relationship from material-level fatigue data. In contrast, effective stress refers to the stress state experienced by the degraded material after stiffness degradation and the resulting local stress redistribution. Thus, although existing progressive damage and residual-property models can describe stiffness or strength degradation, the direct use of a conventional fatigue-life relation does not necessarily ensure consistency between the prescribed stress level used to obtain *N_f_* and the evolving effective stress experienced by a damaged finite-element integration point. This distinction provides the specific research gap addressed in the present study: how to correct the fatigue-life input when the local stress state associated with the fatigue process evolves with material degradation. The underlying hypothesis is that accounting for this evolution can reduce the discrepancy between fatigue-life information obtained from standard tests and its application in finite element fatigue analysis. This hypothesis is examined subsequently through element-level verification and structural experiments rather than assumed a priori.

In addition, an appropriate description of fatigue stiffness degradation over different stress levels is required to implement this correction within a progressive finite element framework. Conventional analytical fatigue stiffness degradation models generally describe the normalized stiffness–life relationship using prescribed functional forms and stress-dependent fitting parameters. While such formulations are useful for individual stress levels, their simple parameterized forms may become less flexible when the degradation behavior changes over multiple stress levels and when experimental data from different fatigue conditions need to be represented simultaneously. An artificial neural network (ANN) therefore provides a data-driven means of mapping stress level and fatigue state to normalized stiffness degradation without imposing a predefined analytical relationship. In the present study, the ANN is used specifically to characterize the relationship among stress level, normalized fatigue life, and normalized fatigue stiffness, thereby making fuller use of the available fatigue experimental data and providing the degradation information required by the progressive damage analysis.

Based on these considerations, this work pursues three main objectives: (1) establish a fatigue-life correction model that accounts for the evolution of the local stress state during fatigue damage accumulation; (2) develop an ANN-based fatigue stiffness degradation model for representing stiffness degradation across different stress levels; and (3) evaluate the resulting fatigue progressive damage framework using flexible composite structures subjected to combined flexural/tensile fatigue loading.

## 2. The Fatigue Progressive Damage Model (FPDM)

The FPDMs mainly include four parts: stress analysis [38], fatigue-life model, fatigue degradation model [39], and failure criterion [40]. The kernel of FPDMs is to predict fatigue life and fatigue property degradation.

### 2.1. Fatigue-Life Correction Model (FLCM)

In this section, a fatigue-life correction model (FLCM) will first be developed by using experimental data obtained by the authors [39]. The material constants of 3232A/Glass are presented in Table 1. Herein, the potential issues of the traditional FLM used in FEM will be clarified. Then, by element fatigue verification, the difference in basic mechanism and concept between the traditional FLM and the proposed FLCM will be carefully demonstrated. Furthermore, a fatigue-life correction procedure is developed by analyzing the shift of the FLM relationship caused by fatigue stiffness degradation and the associated evolution of the local stress state.

#### 2.1.1. The Traditional Fatigue-Life Model

The traditional FLM developed by Shokrieh and Lessard [11] and Harris et al. [41] is applied in fatigue analysis, which can be expressed as

(1) *a* = *f*(1 − *q*)*^u^*(*c* + *q*)*^v^*

where *f*, *u*, and *v* denote material constants, and *a*, *q*, and *c* are parameters characterizing the fatigue conditions. The formulations are described as follows

(2) $$\begin{array}{l} R=\sigma_{\min}/\sigma_{\max},\;\sigma_{a}=\left(\sigma_{\max}-\sigma_{\min}\right)/2,\;\sigma_{m}=\left(\sigma_{\max}+\sigma_{\min}\right)/2\\ \;\;\;\;c=\left[\sigma^{C}\right]/\left[\sigma^{T}\right],\;a=\sigma_{a}/\left[\sigma^{T}\right],\;q=\sigma_{m}/\left[\sigma^{T}\right] \end{array}$$

where *R* denotes the stress ratio. *σ*_min_ and *σ*_max_ denote minimum and maximum stresses. [*σ*^T^] and [*σ*^C^] signify the tension and compression strengths. *σ_m_* and *σ_a_* represent mean stress and stress amplitude.

To simplify Equation (1), Gathercole et al. [42] found that *u* and *v* are very close for glass-fiber-reinforced polymers (GFRPs) and *f* ≈ 1.06. Thus, *u* = *v* in the present work. In addition, the relation between *u* and lg*N_f_* is linear, which can be given by

(3) *u* = *A* + *B*lg*N_f_*

where *A* and *B* are fitting material constants, and *N_f_* denotes fatigue life. The expression of the FLM can be derived by substituting Equation (1) into Equation (3), which is given by

(4) $$\frac{\ln\left(a/f\right)}{\ln\left[\left(1-q\right)\left(c+q\right)\right]}=u=A+\mathrm{Blg}N_{f}$$

To focus the fatigue-life formulation on the fiber-direction response, the explicit fatigue-life relation is calibrated using the fiber-direction T–T and C–C fatigue data. Matrix cracking and delamination are not assigned independent cycle-dependent fatigue-growth laws in the present formulation. The traditional FLM [39] in the fiber direction can be recalled, which is expressed as

(5) $$\begin{array}{l} u=0.9984+0.6390\mathrm{lg}N_{f},\;T\text{-}T\;\mathrm{fatigue}\\ u=0.5944+0.7565\mathrm{lg}N_{f},\;C\text{-}C\;\mathrm{fatigue} \end{array}$$

Once the stress ratio *R* and stress level *S* have been determined, the fatigue life *N_f_* can be calculated by Equation (5). The stress level *S* is identified as the ratio of the applied stress to the corresponding static strength. Thus, *N_ft_* = 3543 when *S* = 75% for T–T fatigue and *N_fc_* = 31,624 when *S* = 85% for C–C fatigue, which agree well with experimental results.

#### 2.1.2. Element Fatigue Verification

**Figure 1:**
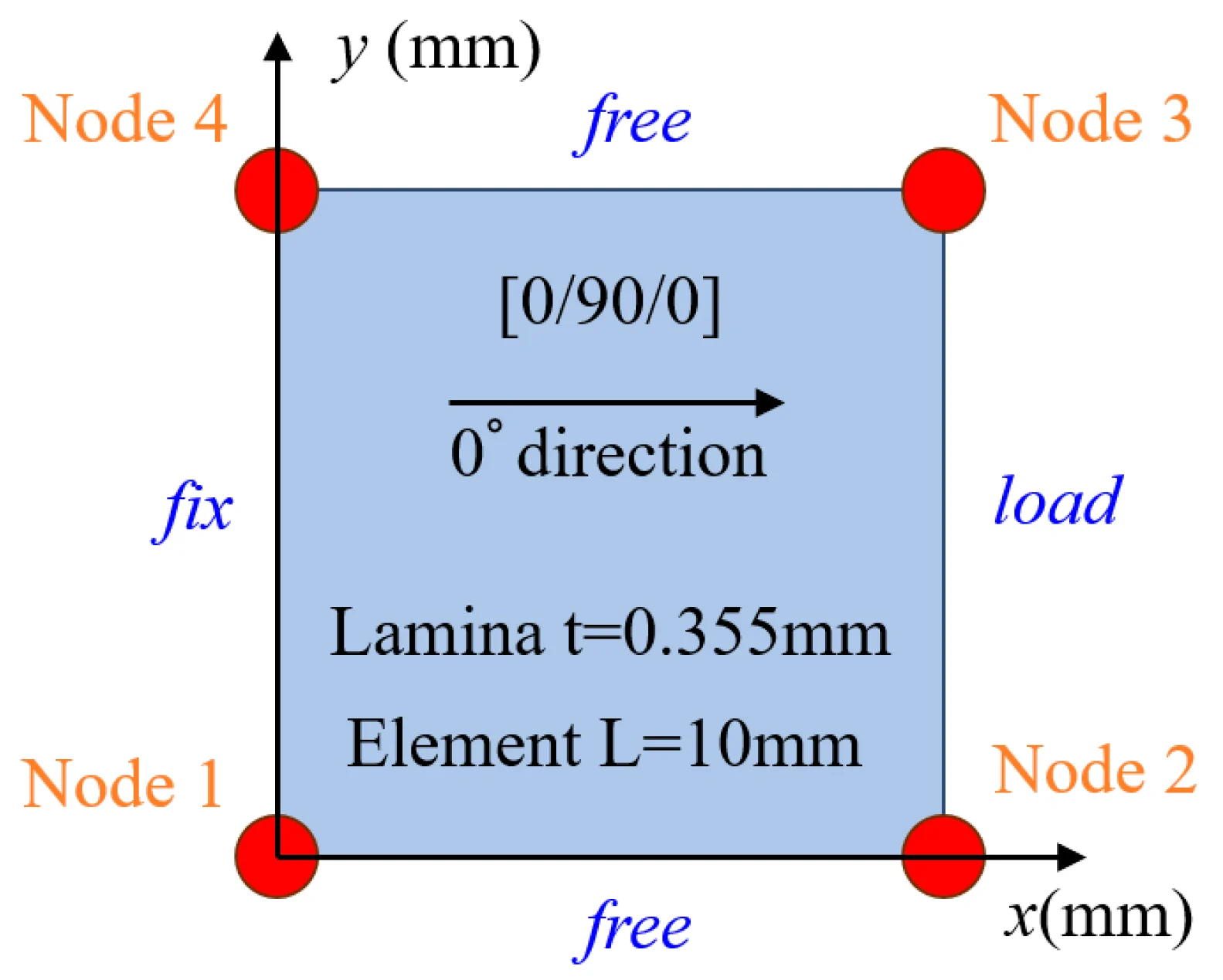
The schematic of element fatigue verification.

To examine the implementation of the traditional FLM in the finite element procedure, an element-level fatigue verification was carried out. The information on the one-element FEM is presented in Figure 1. The element type is S4 in ABAQUS 2019. The length of the element is L = 10 mm and the lamina thickness is t = 0.355 mm. The total thickness of the three-layer plate [0°/90°/0°] is T = 1.065 mm.

**Figure 2:**
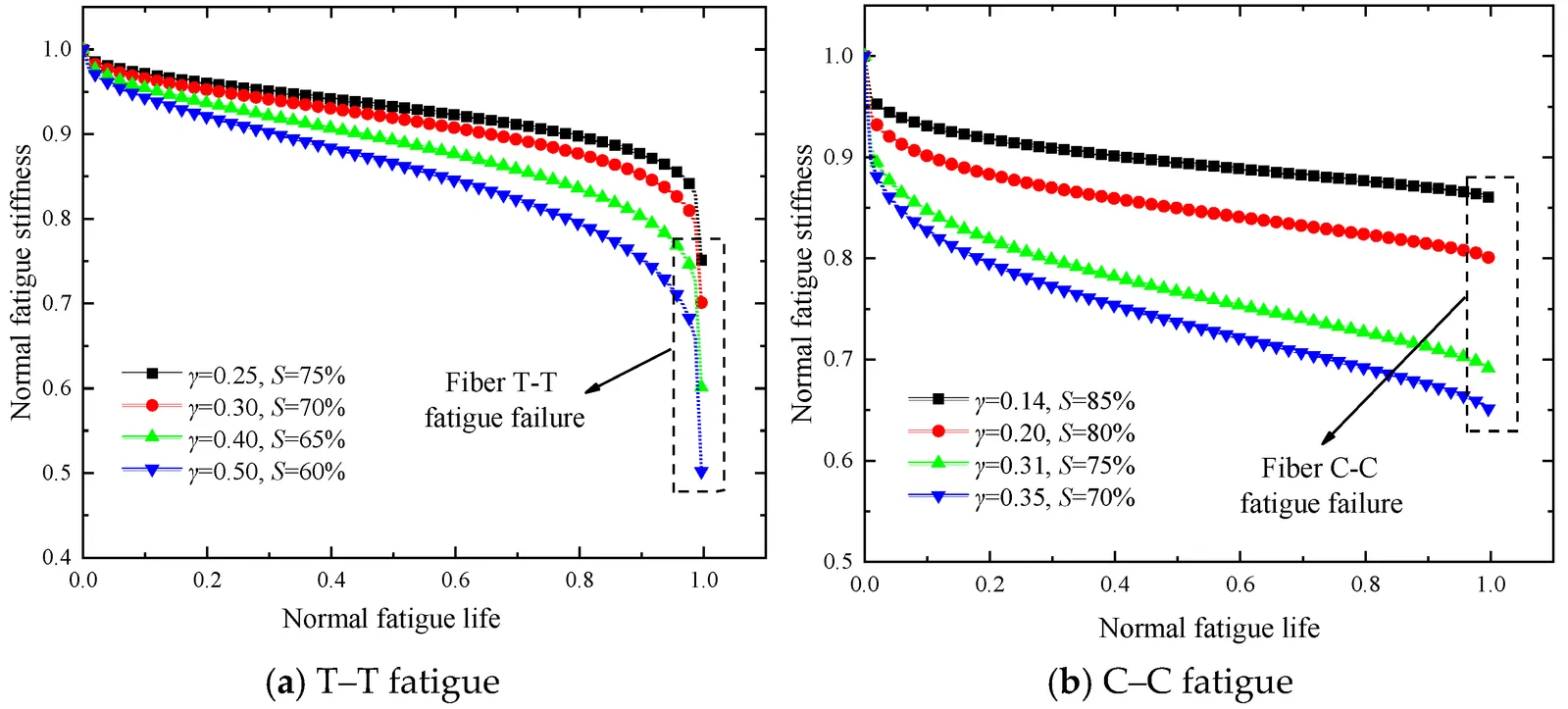
The fitting results of the traditional FSDM.

By fitting experimental data, the traditional fatigue stiffness degradation model (FSDM) [43] at different stress levels can be written as

(6) $$\begin{array}{c} y=1-\gamma \left[1-{\left(1-x^{\alpha }\right)}^{\beta }\right]\\ y=E(n)/E(0),\;x=n/N_{f},\;\gamma =1-E(N_{f})/E(0) \end{array}$$

where *α* and *β* are fitting material constants, and *y* and *x* denote normalized fatigue stiffness and normalized fatigue life, respectively. *γ* represents the decline of fatigue stiffness, which is related to the stress level *S*. The specific fitting results are shown in Figure 2 and the explicit forms of the traditional FSDM are presented by

(7) $$\begin{array}{l} y=1-\gamma \left[1-{\left(1-x^{0.3487}\right)}^{0.2041}\right],\;T\text{-}T\;\mathrm{fatigue}\\ y=1-\gamma \left[1-{\left(1-x^{0.2017}\right)}^{0.6890}\right],\;C\text{-}C\;\mathrm{fatigue} \end{array}$$

The typical Hashin failure criterion [44] was applied to determine whether the element has experienced static strength failure, and the elastic moduli are suddenly reduced according to the failure modes [45], which are shown in Table 2. When the stress level remains below the static failure criterion, the material stiffness is continuously degraded according to the fatigue stiffness degradation model. Once a Hashin failure criterion is satisfied, however, the corresponding element enters a post-failure state and a sudden residual-stiffness reduction is introduced. The residual stiffness factor is used to represent the substantially reduced load-carrying capability after element failure while avoiding a direct reduction to zero stiffness, which may cause numerical instability and severe convergence difficulties during subsequent load redistribution. Similar progressive fatigue damage formulations combine gradual stiffness degradation with sudden material-property degradation associated with failure criteria [3].

In order to accelerate the fatigue process, a single simulation cycle corresponds to a fatigue cyclic jump of 20 cycles (Δ*N* = 20), which satisfies Δ*N* < 0.01*N_ft_* and maintains the precision of the algorithm. In the one-element FEM test, the matrix damage occurred in the static loading phase. Thus, an explicit expression to calculate the external load applied to the element can be given by

(8) $$SX=\sigma_{\max}=\frac{F_{\max}}{Lh^{\mathrm{eq}}}=\frac{F_{\max}}{L\left(2t+\frac{E_{2f}}{E_{1}}t\right)}=\frac{F_{\max}}{A^{\mathrm{eq}}}$$

where *S* and X denote stress level and strength. *σ*_max_ and *F*_max_ denote maximum stress and applied external load. L is the element length in Figure 1. E_1_ and E_2_*_f_* are elastic moduli, which can be found in Table 1 and Table 2. *h^eq^* and *A^eq^* represent the equivalent thickness and area of the element when matrix damage has occurred, and *A^eq^* = 7.2305 mm^2^. Thus, *F_t_*_max_ = 8048 N when *S* = 75% for T–T fatigue and *F_c_*_max_ = 7124 N when *S* = 85% for C–C fatigue.

**Figure 3:**
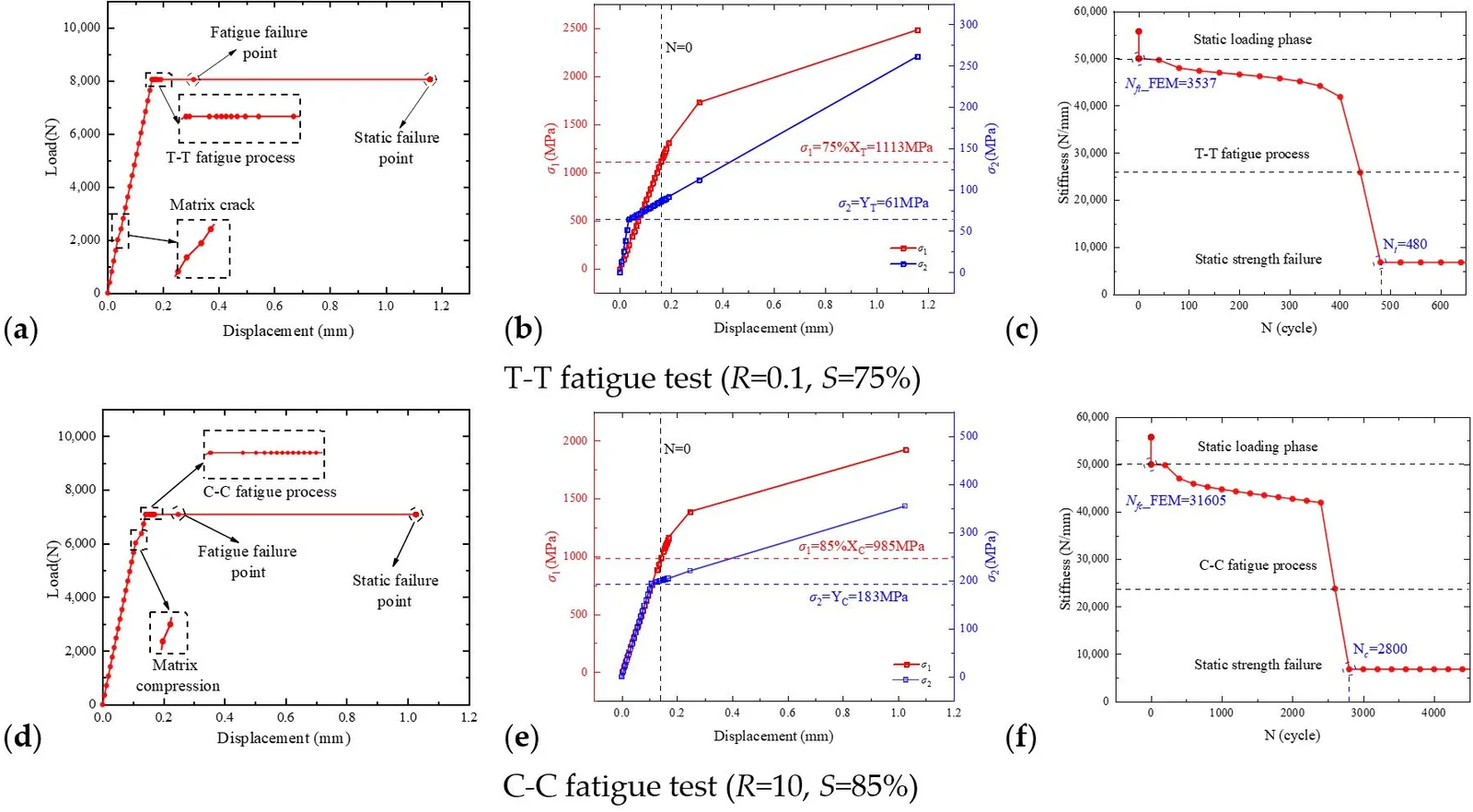
The results of the element FEM in T–T fatigue and C–C fatigue tests. (**a**) Load-Displacement curve of T-T fatigue; (**b**) Stress-Displacement curve of T-T fatigue; (**c**) Stiffness-Cycle curve of T-T fatigue; (**d**) Load-Displacement curve of C-C fatigue; (**e**) Stress-Displacement curve of C-C fatigue; (**f**) Stiffness-Cycle curve of C-C fatigue.

The traditional FLM in Equation (5) and the traditional FSDM in Equation (7) have been implemented into ABAQUS. In the present UMAT implementation, the fatigue-induced material degradation is represented primarily through stiffness degradation, while the strength parameters used in the Hashin failure criteria are assumed to remain constant. Under this assumption, fatigue damage progressively reduces the local load-carrying stiffness and consequently modifies the stress distribution within the structure. The initiation of subsequent Hashin-based failure is then determined by the redistributed local stress state relative to the unchanged strength limits. This treatment simplifies the coupled residual-stiffness/residual-strength evolution and may affect the predicted timing of final damage initiation. Therefore, the present model should be regarded as a stiffness-dominated fatigue damage model, while further incorporation of residual-strength degradation would be an important extension for future work. Then, the results of element fatigue verification in T–T fatigue and C–C fatigue tests are presented in Figure 3, which include Load–Displacement (F–U) curves, Stress–Displacement (*σ*–U) curves, and Stiffness–Life (F/U–N) curves.

From Figure 3, several conclusions can be summarized as follows: (1) In Figure 3a,d, the matrix damage occurred in the static loading phase for T–T and C–C fatigue tests. Therefore, in Figure 3b,c,e,f, the stress and stiffness of the element changed in the static loading phase along with the matrix damage. (2) In Figure 3b,c,e,f, at the time of the initial fatigue process (*N* = 0 cycles), the stress *σ*_1_, *σ*_2_, and *N_f_*_FEM are consistent with the preset stress level *S* in Equation (8) and the fatigue life calculated by Equation (5), which means that the UMAT subroutine can accurately obtain the traditional FLM. In addition, the fatigue process of F/U–N curves in Figure 3c,f verifies that the UMAT subroutine is effective in reproducing the traditional FSDM function in Equation (7). (3) The fatigue life predicted for the element is *N_t_* = 480 cycles in T–T loading and *N_c_* = 2800 cycles in C–C loading, compared with *N_ft_* = 3543 and *N_fc_* = 31,624 cycles obtained from the traditional FLM, respectively. The corresponding differences indicate that the stress–fatigue-life relationship used by the conventional FLM is shifted during the fatigue analysis.

#### 2.1.3. Definition of the Fatigue-Life Discrepancy

To visually demonstrate the ‘shift phenomenon’ of the traditional FLM in FEM, the T–T element fatigue verifications were carried out at *S* = 60%, 65%, 70%, and 75%, and the C–C element fatigue verifications were carried out at *S* = 70%, 75%, 80%, and 85%. The corresponding external load *F*_max_ and the decline of fatigue stiffness *γ* can be obtained from Equation (8) and Figure 2.

**Figure 4:**
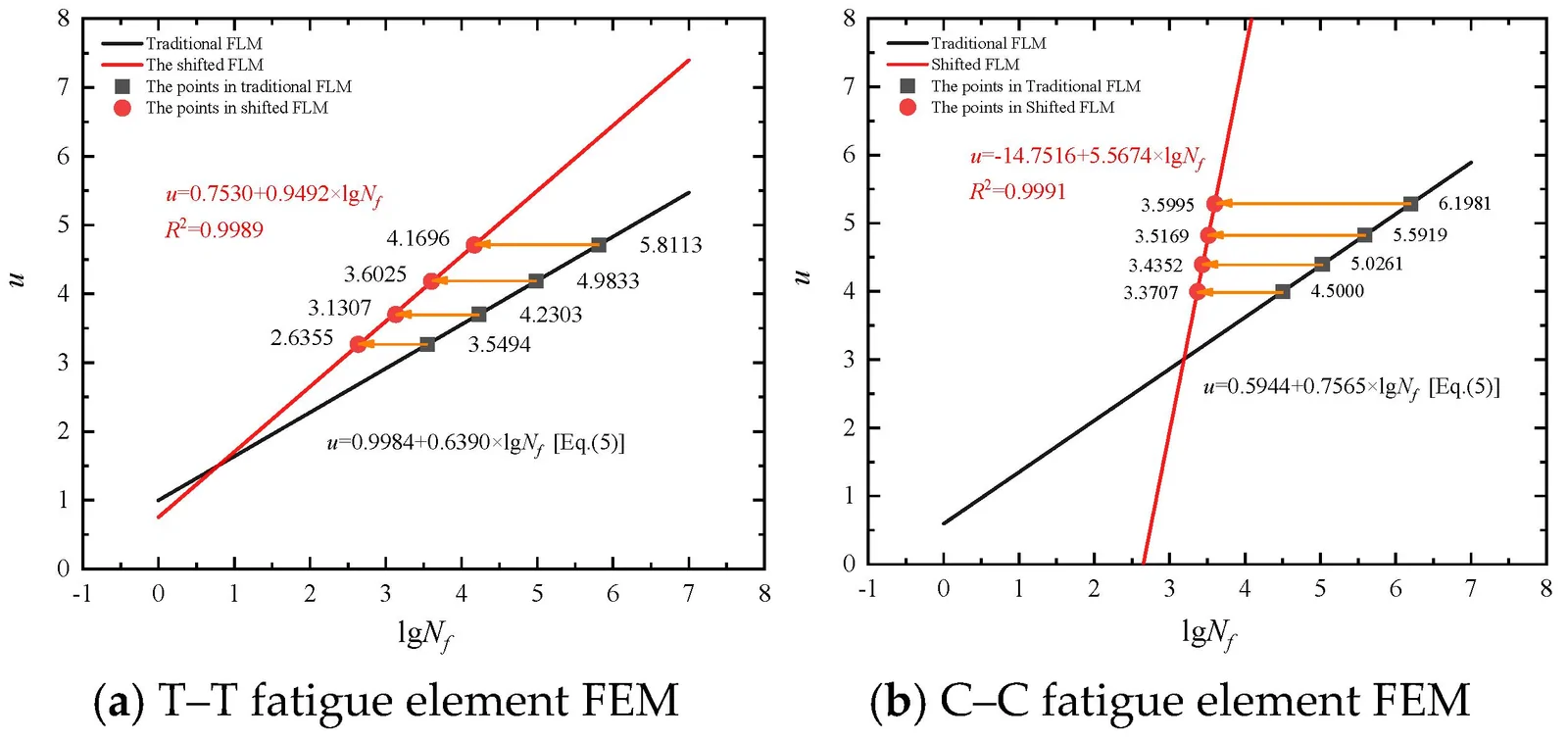
The shifted FLM and traditional FLM in element fatigue verification (*γ* is variable).

The fatigue cyclic jump is Δ*N* = 1, which means that the algorithm has no error due to Δ*N*. Then, the shifted FLM and traditional FLM in element fatigue verification (*γ* is variable) are shown in Figure 4. It is obvious that the shifted points still satisfy the linear relationship of Equation (3). Therefore, the shifted points have been re-fitted by Equation (3). After T–T and C–C element fatigue verification in FEM, it can be found that the predicted fatigue life in the shifted FLM function (red line and points) is far away from the fatigue life in the traditional FLM function (black line and points).

**Figure 5:**
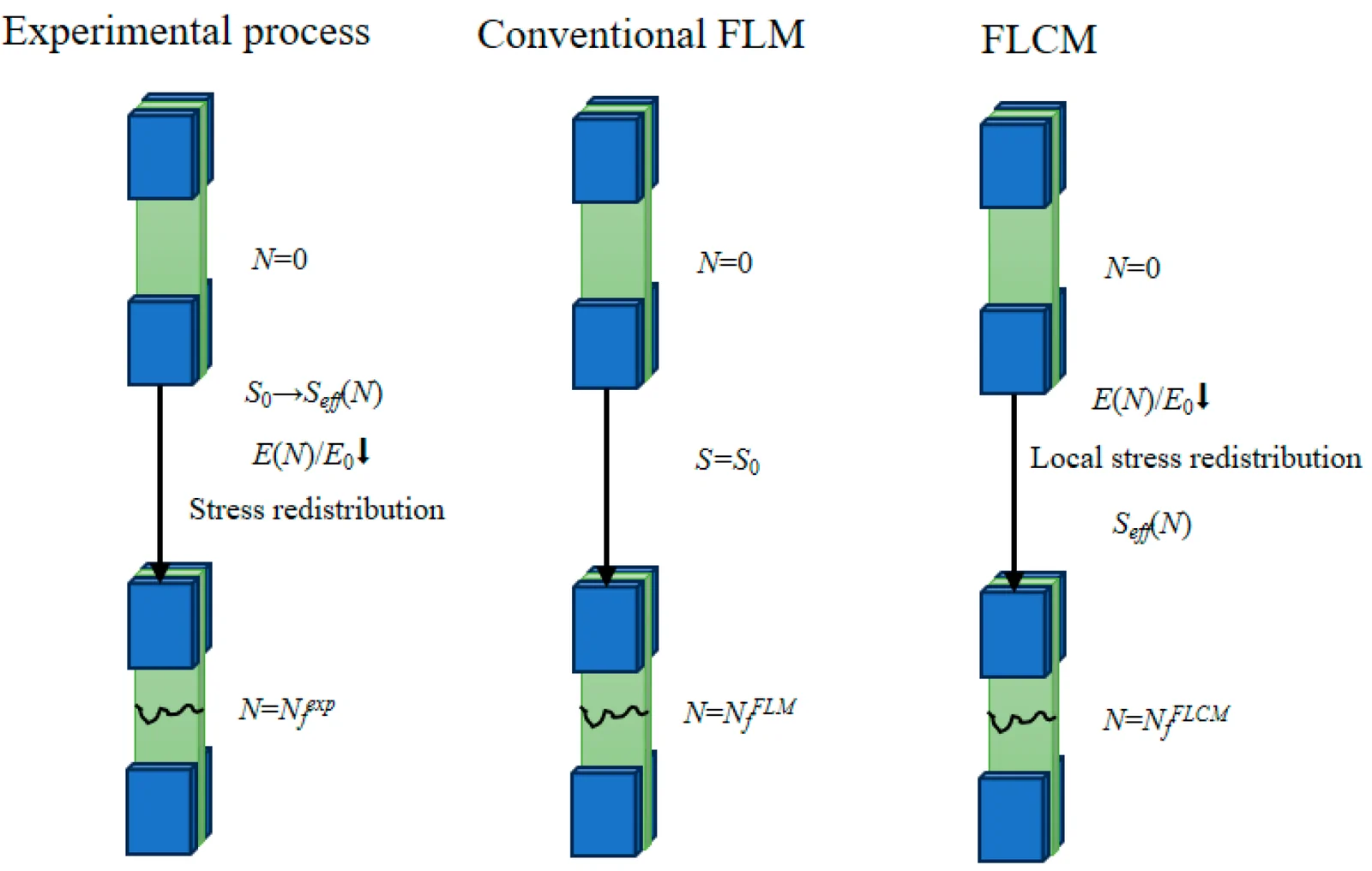
Schematic illustration of the fatigue-life discrepancy caused by the evolution of the local stress state during fatigue loading.

To clarify the difference between the fatigue life obtained from experiments and that used in the conventional fatigue analysis, three quantities are distinguished. The experimental fatigue life, denoted by $N_{f}^{\exp}$, is the number of cycles from the prescribed initial loading condition to the experimentally observed fatigue failure. The fatigue life obtained from the conventional FLM, denoted by $N_{f}^{\mathrm{FLM}}$, is calculated by Equation (5) using the initial prescribed stress level *S*_0_ as a constant stress level throughout the fatigue process. The fatigue life obtained after introducing the proposed FLCM is denoted by $N_{f}^{\mathrm{FLCM}}$. The schematic illustration of the fatigue-life discrepancy caused by the evolution of the local stress state during fatigue loading is shown in Figure 5.

For the element-level analysis, the initial local stress level is defined by the local stress at the beginning of fatigue loading, *S*_0_ = *σ_local_*_,0_/*X*, where *X* denotes the corresponding static strength. During cyclic loading, fatigue stiffness degradation changes the local structural stiffness and consequently causes stress redistribution. Therefore, the local stress level is generally a function of fatigue cycles, which can be expressed conceptually as *S_eff_*(*N*) = *σ_local_*(*N*)/*X*. Thus, the essential difference between the conventional FLM and the proposed FLCM is that the former evaluates fatigue life using the prescribed initial stress level *S*_0_, whereas the latter accounts for the evolution of the effective local stress state associated with fatigue stiffness degradation.

For the 0° T–T fatigue case with *R* = 0.1 and *S*_0_ = 75%, the conventional FLM gives *N_ft_* = 3543 cycles, corresponding to an initial stress of 1113 Mpa. The element-level FEM result gives *N_t_* = 480 cycles. The corresponding C–C case gives *N_fc_* = 31,624 cycles from the conventional FLM and *N_c_* = 2800 cycles from the element FEM analysis. These results provide a measurable basis for identifying the shift of the FLM relationship during fatigue analysis.

The conventional FLM and element-level FEM produce substantially different fatigue-life predictions. Therefore, it is necessary to establish the fatigue-life correction model (FLCM) in fatigue FEM. The FLCM is required to reproduce the traditional FLM by considering the ‘shift phenomenon’.

#### 2.1.4. The Mechanism and Construction of the FLCM

In this section, the development of the proposed FLCM is discussed. First, to investigate the origin of the shift phenomenon, the fatigue-stiffness-degradation parameter *γ* is temporarily fixed. Under a fixed *γ*, the fatigue stiffness degradation follows the same prescribed degradation trajectory, allowing the effect of the evolving local stress state on the FLM relationship to be examined separately from the variation of *γ*.

**Figure 6:**
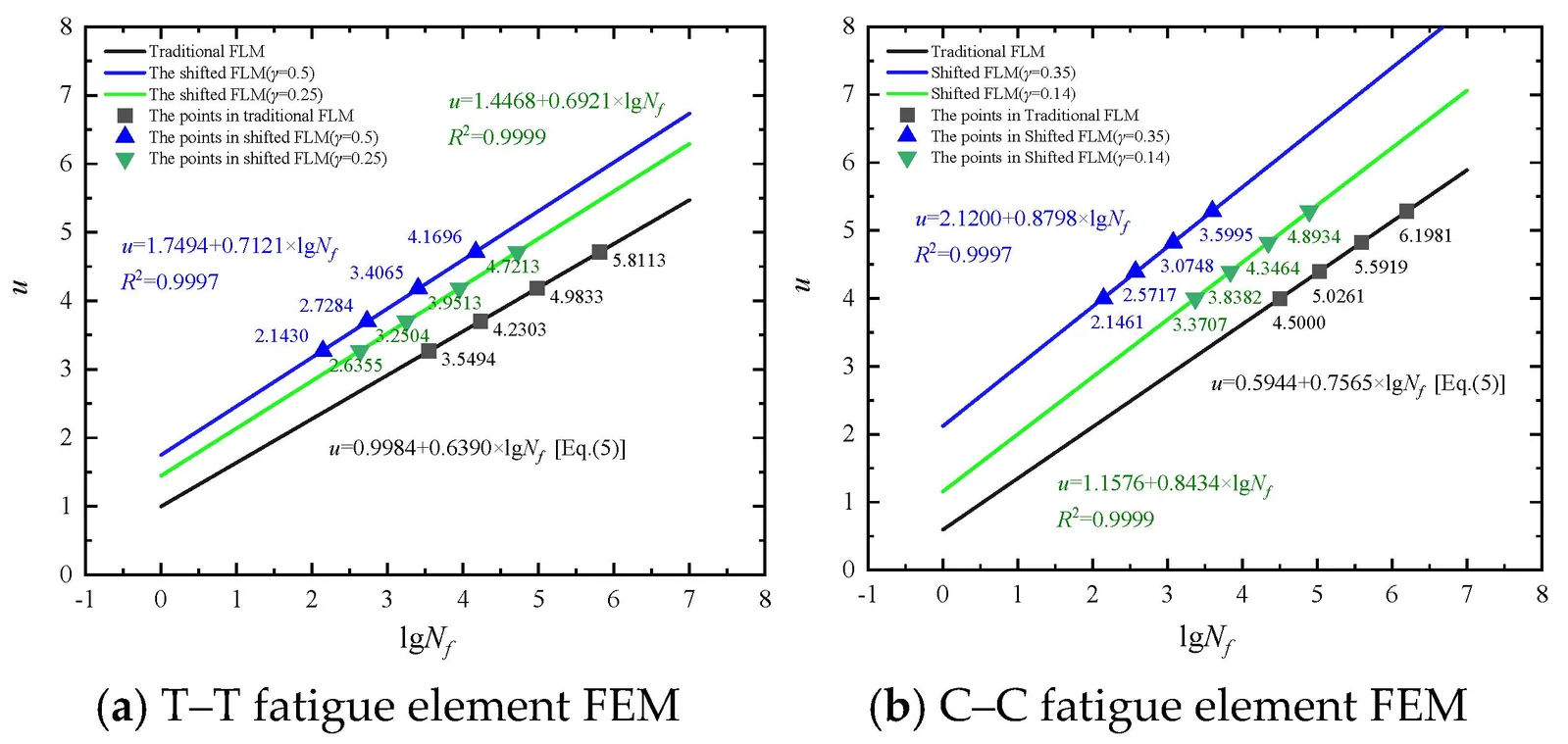
Traditional and shifted FLM relationships under fixed fatigue-stiffness-degradation parameters: (**a**) T–T loading; (**b**) C–C loading.

Then, the shifted FLM and traditional FLM obtained from the element-level FEM analysis under constant *γ* are shown in Figure 6. It can be observed that the shifted data points remain highly linear and still satisfy the linear relationship described by Equation (3).

To quantitatively characterize the fitted linear relationships, the fitted slope *B*, coefficient of determination (*R*^2^), and the corresponding 95% confidence interval (CI) of *B* are summarized in Table 3. The shifted relationships exhibit high coefficients of determination (*R*^2^ > 0.9996), indicating that the linear form of the conventional FLM is retained after the shift.

As shown in Table 3, the fitted slopes of the shifted relationships exhibit moderate deviations from those of the traditional FLM. Therefore, the slope variation is not used as an additional fitting parameter in the present model. Instead, *B* is retained as a fixed parameter under a given loading condition, while the shift caused by fatigue stiffness degradation and the associated evolution of the local stress state is represented by the change in the intercept *A*. This treatment preserves the original functional form of the FLM while introducing the minimum additional parameter required to describe the shift phenomenon.

Accordingly, under the working assumption that *B* remains unchanged for a fixed *γ*, Equation (5) can be rewritten as Equation (9), in which the intercept *A* is subsequently determined from the shifted fatigue response.

The use of a fixed *B* is intended to retain the stress–fatigue-life dependence established by the conventional FLM and to avoid introducing an additional slope parameter for each fatigue-stiffness-degradation condition. Thus, the effect of the shift phenomenon is incorporated through the correction of *A*, while *B* preserves the original FLM dependence on fatigue life.

(9) $$\begin{array}{l} u=A+0.6390\mathrm{lg}N_{f},\;T\text{-}T\;\mathrm{fatigue}\\ u=A+0.7565\mathrm{lg}N_{f},\;C\text{-}C\;\mathrm{fatigue} \end{array}$$

Second, the fitting material constant *A* in Equation (9) is determined by a bisection procedure for each prescribed stress level *S* and fixed fatigue-stiffness-degradation parameter *γ*. For a given *S* and *γ*, the objective is to determine the value of *A* that enables Equation (9) to reproduce the corresponding fatigue-life response of the conventional FLM.

Specifically, the residual function is defined as the difference between the fatigue-life response calculated from Equation (9) and the target fatigue-life response of the conventional FLM at the same stress level. Two initial bounds, *A*_L_ and *A*_U_, are selected such that the residuals at the two bounds have opposite signs, i.e., *g*(*A*_L_)*g*(*A*_U_) < 0. The midpoint is then calculated as *A*_M_ = (*A*_L_ + *A*_U_)/2, and the corresponding fatigue-life response is obtained from Equation (9). According to the sign of the residual at *A*_M_, either *A*_L_ or *A*_U_ is replaced by *A*_M_, while the other bound is retained. The interval is therefore progressively reduced while maintaining the bracketing condition. This procedure is repeated until the calculated fatigue-life response converges to the target value to the required numerical precision. In the present calculation, the iterative process was practically terminated when the corresponding lg*N_f_* value converged to approximately four decimal places.

The initial bounds are selected from the numerical range in which the target fatigue-life response is bracketed; therefore, their selection provides the starting interval for the numerical search rather than an additional material parameter. The bisection procedure itself is deterministic once the initial bracketing interval and convergence criterion are specified. Because the residual changes monotonically within the selected bracketing interval in the present calculations, the converged solution is uniquely determined within that interval. The obtained *A* is subsequently used to construct the FLCM for the specified *S* and *γ*.

The numerical implementation uses the fatigue-life response calculated at each iteration rather than directly fitting *A* from the target point. Therefore, the bisection procedure determines the intercept required for the corrected formulation to recover the corresponding reference fatigue-life response.

Then, the average value of *A* obtained at the investigated stress levels is calculated and substituted into Equation (9) to construct the FLCM under a fixed *γ*. The parameters and fatigue-life results involved in the construction of the FLCM under fixed *γ* are presented in Table 4. Here, *A* is the newly determined correction parameter for the fixed-*γ* formulation, whereas *S*, *γ*, and the parameters of the conventional FLM are obtained from the corresponding fatigue stiffness and fatigue-life relations. The sources and roles of the main mathematical quantities used in the conventional FLM and the proposed FLCM are further summarized in Table 5.

**Figure 7:**
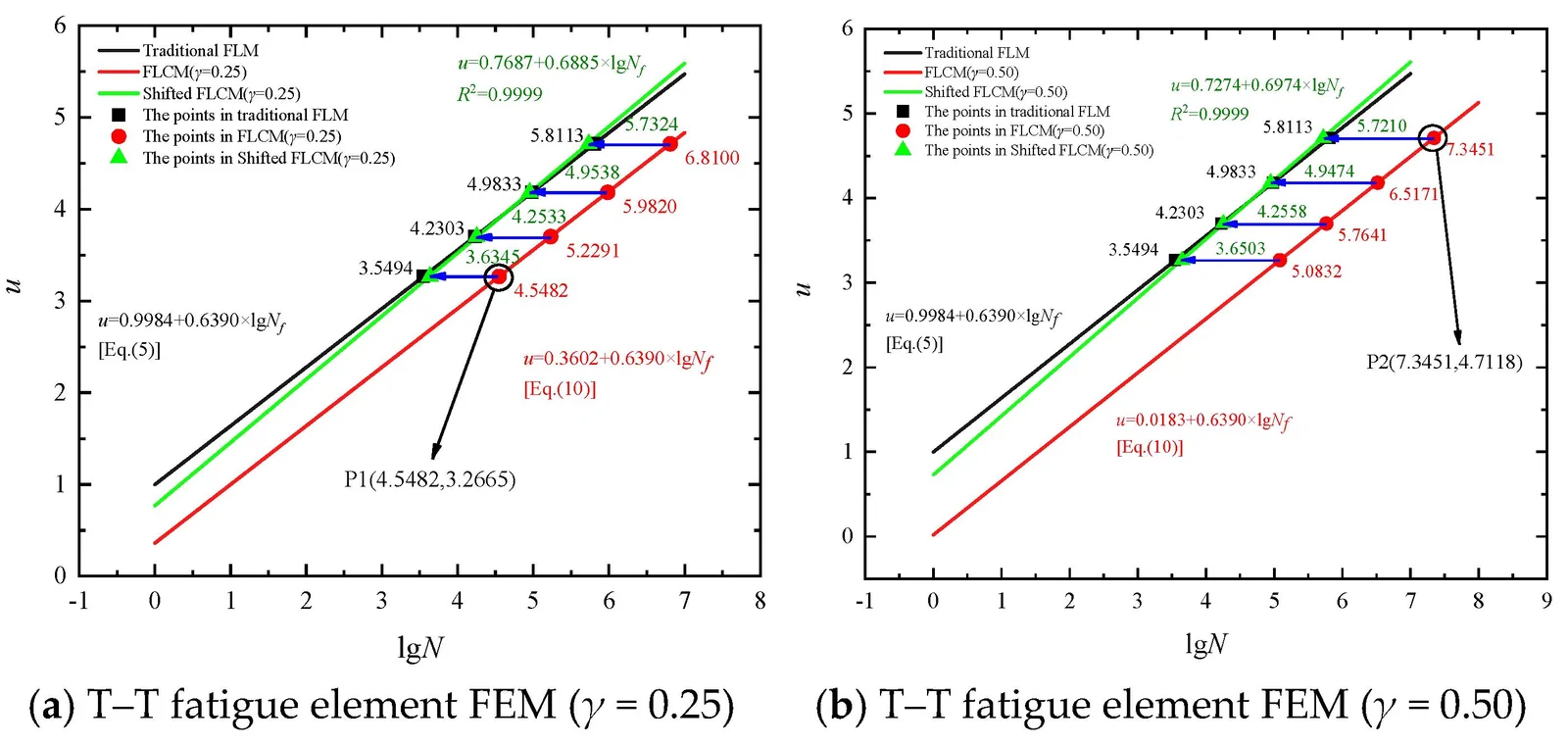

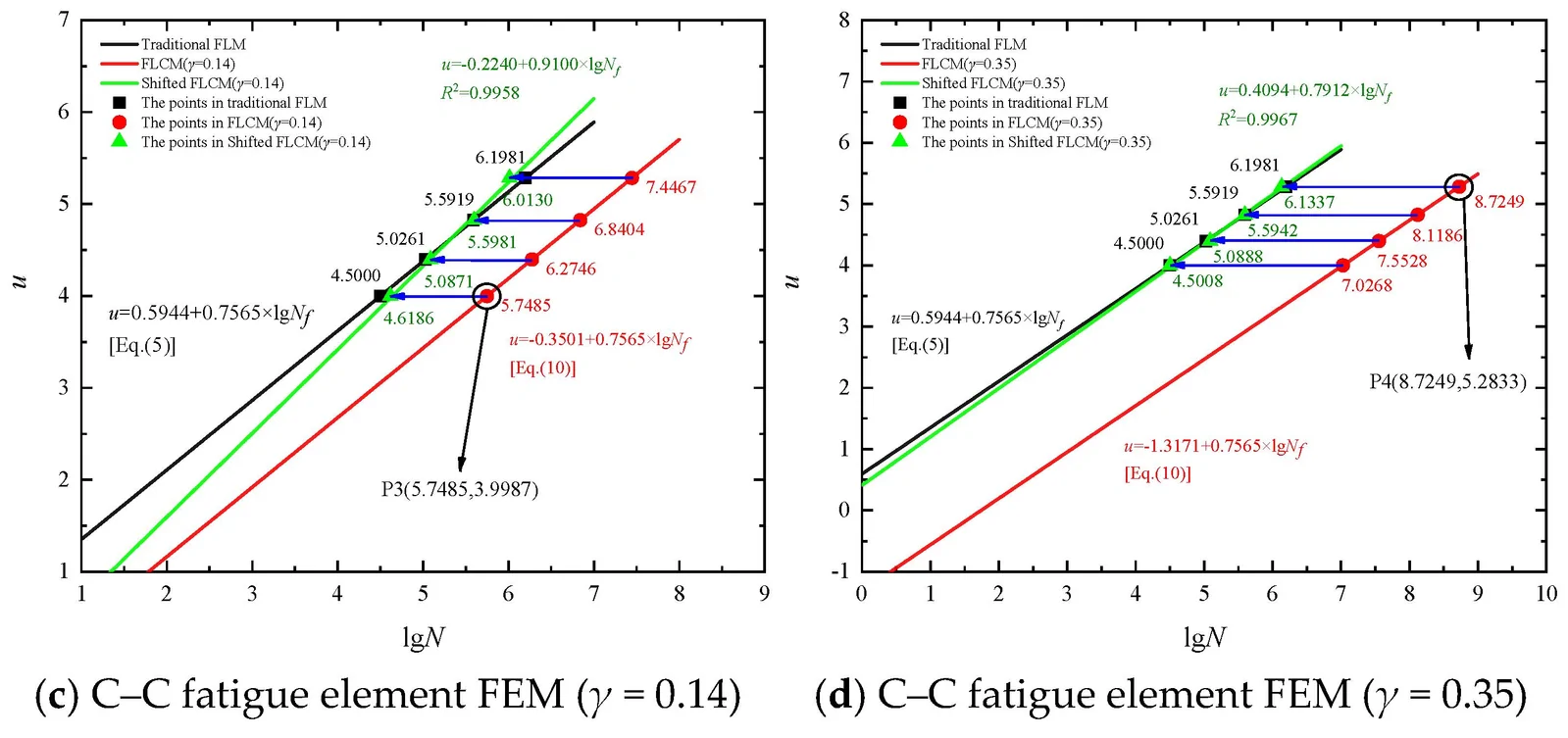
The FLCM and traditional FLM in the element FEM test (*γ* is constant).

The FLCM and traditional FLM obtained from the element-level fatigue analysis under fixed *γ* are compared in Figure 7. Based on the determined *A*, Equation (9) is updated to Equation (10). The results show that the corrected formulation closely reproduces the reference fatigue-life relationship of the traditional FLM over the investigated stress levels. In particular, the reconstructed relationship remains consistent with the linear form of the conventional FLM within the investigated range. This comparison is regarded as an internal consistency verification of the correction procedure rather than an independent validation, because the parameter *A* is determined using the corresponding reference fatigue-life response. Independent validation of the fatigue-life prediction is provided by the structural fatigue experiments presented in Section 4.

(10) $$\begin{array}{l} \left\{\begin{array}{c} u=0.3602+0.6390\mathrm{lg}N_{f},\;\gamma =0.25\\ u=0.0183+0.6390\mathrm{lg}N_{f},\;\gamma =0.50 \end{array}\right.\;T\text{-}T\;\mathrm{fatigue}\\ \left\{\begin{array}{c} u=-0.3501+0.7565\mathrm{lg}N_{f},\;\gamma =0.14\\ u=-1.3171+0.7565\mathrm{lg}N_{f},\;\gamma =0.35 \end{array}\right.\;C\text{-}C\;\mathrm{fatigue} \end{array}$$

**Figure 8:**
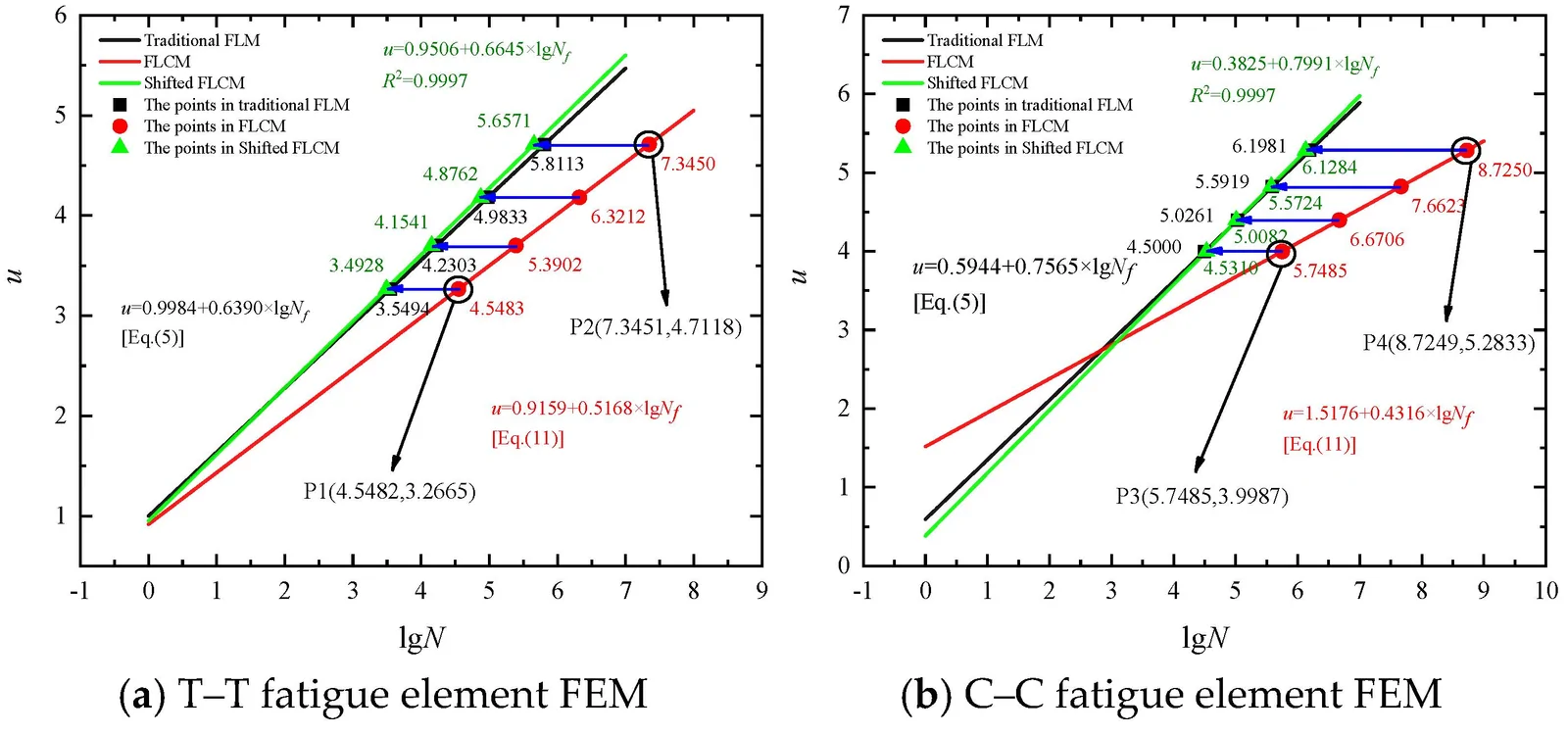
The FLCM and traditional FLM in the element FEM test (*γ* is variable).

Third, to extend the fixed-*γ* formulation to the investigated range of stress levels, *γ* is allowed to vary according to the fatigue stiffness degradation data in Figure 2. The T–T and C–C FLCM relationships are then constructed using the corresponding characteristic points P1–P4, as shown in Figure 7. The FLCM and traditional FLM in the element FEM test (*γ* is variable) are presented in Figure 8. Thus, Equation (10) can be updated as Equation (11). The relative discrepancy between the fatigue-life predictions of Equation (11) and the conventional FLM is evaluated using the logarithmic fatigue-life error defined in Table 4. The maximum discrepancy over the investigated stress levels is below 6%. This result demonstrates the internal consistency of the proposed correction formulation over the Investigated range rather than serving as an independent validation of the model. Independent validation of the complete fatigue progressive damage model is performed using the structural fatigue experiments in Section 4.

(11) $$\begin{array}{l} u=0.9159+0.5168\mathrm{lg}N_{f},\;T\text{-}T\;\mathrm{fatigue}\\ u=1.5176+0.4316\mathrm{lg}N_{f},\;C\text{-}C\;\mathrm{fatigue} \end{array}$$

### 2.2. Fatigue Stiffness Degradation Model Based on ANN (FSDM-ANN)

In the present study, the fatigue progressive damage model (FPDM) consists of two coupled material-level components: the fatigue-life correction model (FLCM) and the fatigue stiffness degradation model based on ANN (FSDM-ANN). The FLCM provides the fatigue life *N_f_* corresponding to the current stress state, while the FSDM-ANN determines the evolution of the normalized residual stiffness *E*/*E*_0_ according to the stress level *S* and normalized fatigue life *n*/*N_f_*. The FLCM is newly improved in the present study, whereas the FSDM-ANN is adopted from our previous material-level study [39] and incorporated into the present FPDM. The essential formulation and implementation details are provided below to make the present model self-contained, while avoiding duplication of the complete methodology reported in study [39].

The FSDM-ANN is established exclusively from standard coupon-level experimental data. The subsequent structural-level validation is performed using independent structural specimens, which are not included in the training or validation datasets used to establish the material response surface.

**Figure 9:**
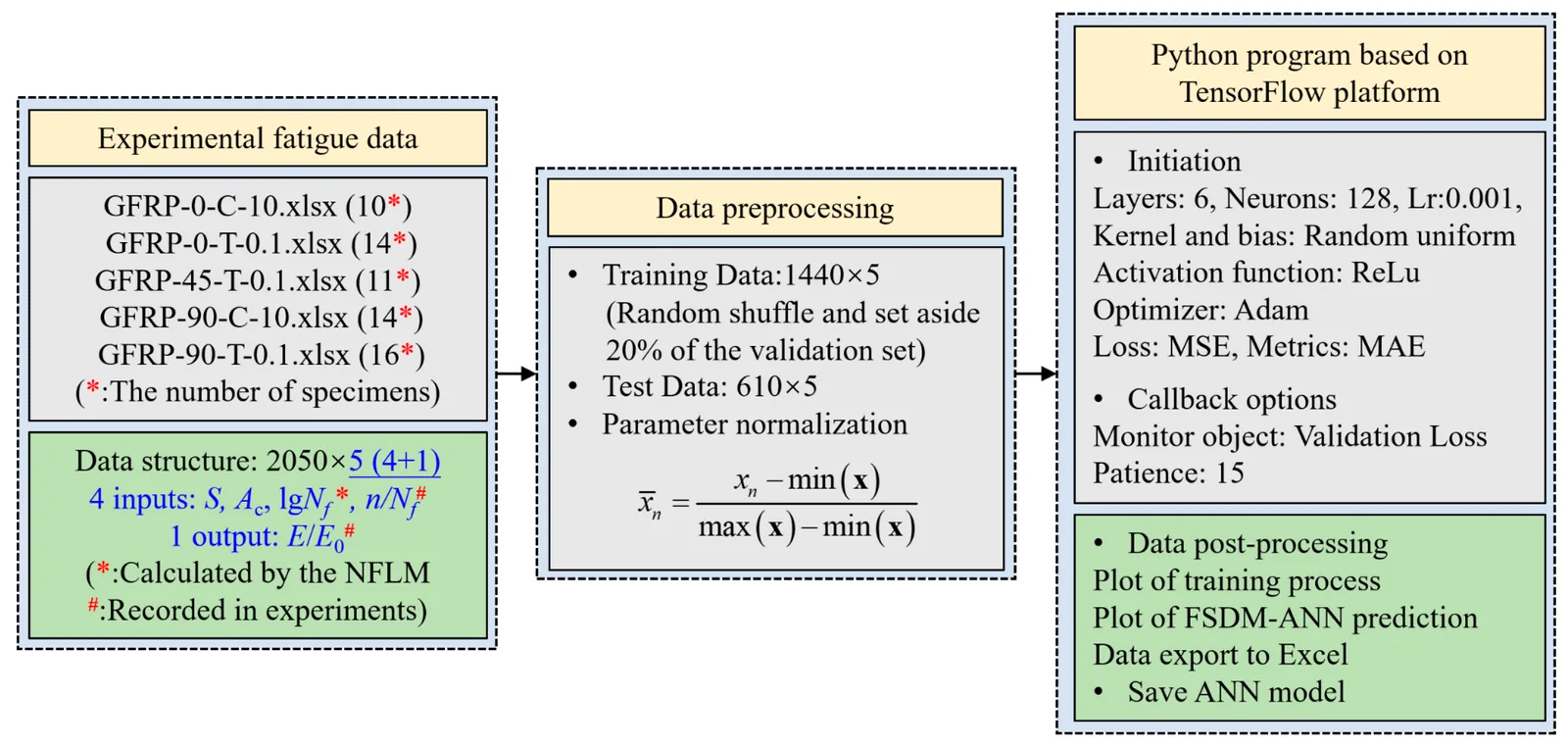
Training process of the FSDM-ANN [39].

For the FSDM-ANN, the input variables are the stress level *S*, cross-sectional area A_c_, logarithmic fatigue life lg*N_f_*, and normalized fatigue life *n*/*N_f_*, while the output variable is the normalized residual stiffness *E*/*E*_0_. For the modified FSDM-ANN employed in the present FPDM, A_c_ is removed because the response surface is established for the same material system. The selected ANN architecture contains six hidden layers with 128 neurons in each hidden layer. The mean squared error (MSE) is used as the loss function. The network is trained using the TensorFlow framework, and the validation loss is monitored during training. An early-stopping criterion with a patience of 15 iterations is adopted to terminate training when no further improvement in the validation loss is obtained. The optimizer, learning rate, batch size, and maximum number of epochs were kept identical to those used in the original implementation [39]. The schematic of the training process of the FSDM-ANN is presented in Figure 9.

The material-level ANN database was constructed exclusively from the standard coupon experiments. The experimental database consists of 25 static specimens and 78 fatigue specimens of GFRP 3232A/Glass. The static tests were used to determine the basic material properties, whereas the fatigue tests provided the stiffness degradation data. For the original FSDM-ANN, 65 valid fatigue specimens were selected, generating 2050 data points with five variables. Among them, 46 specimens were used for model development, from which 20% of the corresponding data points were randomly selected as the validation set, while the remaining 19 specimens were retained as the test set.

For the modified FSDM-ANN used in the present FPDM, 14 standard 0° T–T fatigue specimens and six groups of boundary/empirical data were used, resulting in 600 data points. After row-wise shuffling, 20% of the data were used for validation and the remaining data were used for training. All ANN variables were normalized according to the normalization procedure established for the material-level fatigue model [39].

It should be emphasized that these coupon-level data were used only to establish the material constitutive response surface. None of the structural-level experimental data used for subsequent validation were included in the ANN training or validation datasets. Therefore, the structural validation represents an independent application of the established material model rather than a reuse of the structural experimental data for model calibration.

**Figure 10:**
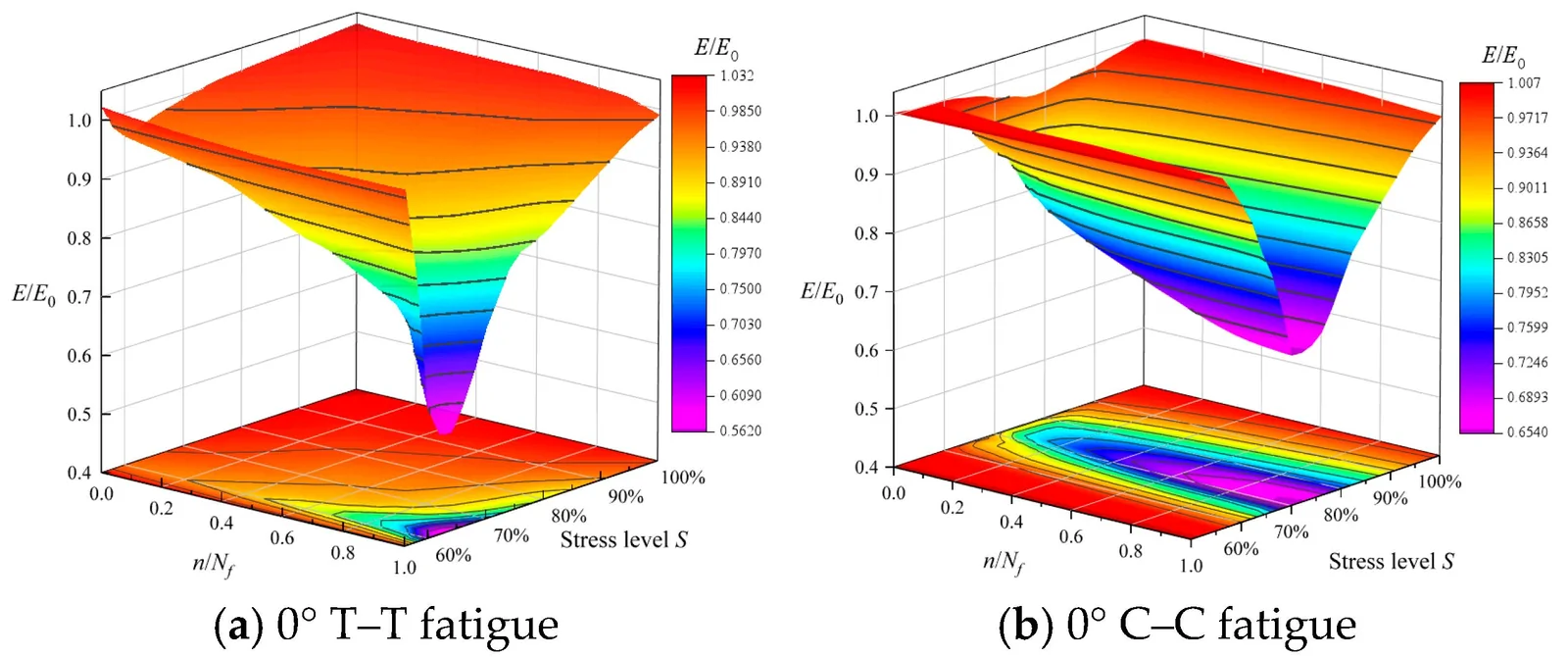
The response surfaces of fatigue stiffness obtained by training the FSDM-ANN.

Traditional FSDM functions in Equation (7) have difficulty characterizing fatigue stiffness degradation over a wide range of stress levels. To overcome the limitations and make full use of fatigue experimental data, the response surfaces of normalized fatigue stiffness for 0° T–T fatigue and 0° C–C fatigue have been trained by the FSDM-ANN [39], which are presented in Figure 10a,b.

The quantitative performance of the ANN response surfaces was further evaluated using the coefficient of determination and the training/validation loss. For the T–T response surface, R2 = 0.9263 and the maximum error is 5.97%, while the training and validation MSE losses are 7.55 × 10^−4^ and 9.85 × 10^−4^, respectively. For the C–C response surface, R2 = 0.9071 and the maximum error is 7.42%, while the training and validation MSE losses are 9.28 × 10^−4^ and 1.13 × 10^−3^, respectively. The relatively close training and validation losses indicate that the response surfaces maintain consistent predictive behavior between the model-development and validation datasets.

In this paper, the proposed FLCM and the FSDM-ANN are implemented into ABAQUS through a UMAT subroutine. During the UMAT calculation, the current stress state at each integration point is first obtained from the finite element analysis, and the corresponding fatigue life *N_f_* is calculated using the FLCM. Consequently, the current stress level *S* and normalized fatigue life *n*/*N_f_* are available at each integration point. These two state variables are then used to query the FSDM-ANN response surface, from which the corresponding residual stiffness ratio *E*/*E*_0_ is obtained.

Because fatigue damage is irreversible, the instantaneous ANN-predicted stiffness ratio cannot be directly used to update the material stiffness when stress redistribution occurs. In particular, after local damage develops, stress redistribution may reduce the current stress level to a value below the fatigue-limit boundary. Directly querying the response surface under such a reduced stress level could result in an artificial recovery of *E*/*E*_0_, which is physically inadmissible.

Therefore, an irreversible damage correction is introduced into the constitutive update. For each integration point, the effective normalized stiffness ratio is defined as

$${\left(\frac{E}{E_{0}}\right)}_{\mathrm{eff}}^{n}=\min\left[{\left(\frac{E}{E_{0}}\right)}_{\mathrm{eff}}^{n-1},{\left(\frac{E}{E_{0}}\right)}_{\mathrm{ANN}}^{n}\right]$$

where ${\left(E/E_{0}\right)}_{\mathrm{ANN}}^{n}$ is the normalized stiffness ratio predicted by the ANN at the current stress state and fatigue-cycle state, and ${\left(E/E_{0}\right)}_{\mathrm{eff}}^{n-1}$ is the minimum normalized stiffness ratio accumulated in the previous loading history. Thus, the current constitutive stiffness is always controlled by the historical minimum value. Once fatigue damage has occurred, subsequent stress redistribution cannot restore the material stiffness, even if the current stress level becomes lower than the fatigue-limit stress level.

For efficient implementation in the UMAT, the continuous response surface is discretized at 1% stress-level intervals over the prescribed stress domain. The resulting response surface is stored as a numerical matrix and directly queried during the constitutive update. When the current state does not coincide with a predefined grid point, the surrounding grid points are used to obtain the corresponding *E*/*E*_0_ by bilinear interpolation.

To prevent unrestricted extrapolation, the response surface is bounded by the physical fatigue-life domain *N_f_* = 1 to *N_f_* = 10^7^. The upper boundary *N_f_* = 1 corresponds to the static-strength condition (*S* = 100%), whereas the lower boundary *N_f_* = 10^7^ corresponds to the fatigue-limit condition. The corresponding fatigue-limit stress level is obtained either directly from the standard fatigue tests or from the fatigue-life model. When the current stress level falls below the fatigue-limit boundary, no additional fatigue stiffness degradation is introduced. Therefore, the UMAT does not require ANN extrapolation outside the prescribed stress–life domain.

Accordingly, the constitutive update procedure of the proposed FPDM can be summarized as follows. First, the finite element analysis provides the current stress state at each integration point. Second, the proposed fatigue-life model calculates the corresponding fatigue life *N_f_*, based on which the stress level S and normalized fatigue life *n*/*N_f_* are determined. Third, *S* and *n*/*N_f_* are used to query the discretized ANN response surface and obtain the instantaneous normalized stiffness ratio ${\left(E/E_{0}\right)}_{\mathrm{ANN}}$. Fourth, the irreversible damage rule is applied by retaining the historical minimum value of *E*/*E*_0_. Finally, the updated stiffness is returned to the finite element solver for the next constitutive iteration. This procedure is repeated at each integration point throughout the fatigue loading process.

## 3. Experiments

### 3.1. Preparation

#### 3.1.1. The Flexible Composite Structures (FCS)

The FCS consists of a metal bush, filling block, and winding prepreg. The configuration and materials of different parts of the structure are presented in Table 6. The FCS specimens were manufactured and supplied by an aerospace manufacturing organization. The curing cycle consisted of heating to 80 °C and holding for 2 h, followed by heating to 120 °C and holding for 2.5 h, and subsequently cooling to room temperature. It should be noted that the FCS tests in this study are intended primarily for independent structural-level validation of the proposed material-level FPDM rather than for establishing a comprehensive manufacturing–fatigue relationship.

In order to capture the stress distribution of the specimen in experiments, it is important to lay out strain gauges reasonably. Strain gauges were installed along the winding/fiber direction, as specified in Table 6. The upper and lower surfaces of the FCS are equipped with 10 strain gauges (BE120-3AA(11)-P15 from AVIC Electronics Measurement). Section numbers 1–6 correspond to the center line of the metal bush, the fixed end of the fixture, and four critical sections. The strain signals were acquired at a sampling frequency of 5 Hz during the mechanical tests.

Four FCSs are prepared, in which two FCSs are used for the static experiment and the other two FCSs are used for the fatigue experiment. The experimental conditions are presented in Table 7. In the static experiment, one side of the structure is fixed, and the other side of the structure is subjected to flexural load until failure. In the fatigue experiment, the structure is subjected to flexural cyclic displacement and constant tensile load. The initial flexural stress level is determined by the ultimate failure load in the static experiment, and the maximum of the flexural cyclic displacement is obtained by calibration of strain gauges. Due to the displacement loading mode, the fatigue damage rate of the specimen gradually decreases until propagation of damage stops. Therefore, it is necessary to increase the maximum of the flexural cyclic displacement to recalibrate the flexural stress level.

#### 3.1.2. The Setup of the Fixture and Testing Machine

**Figure 11:**
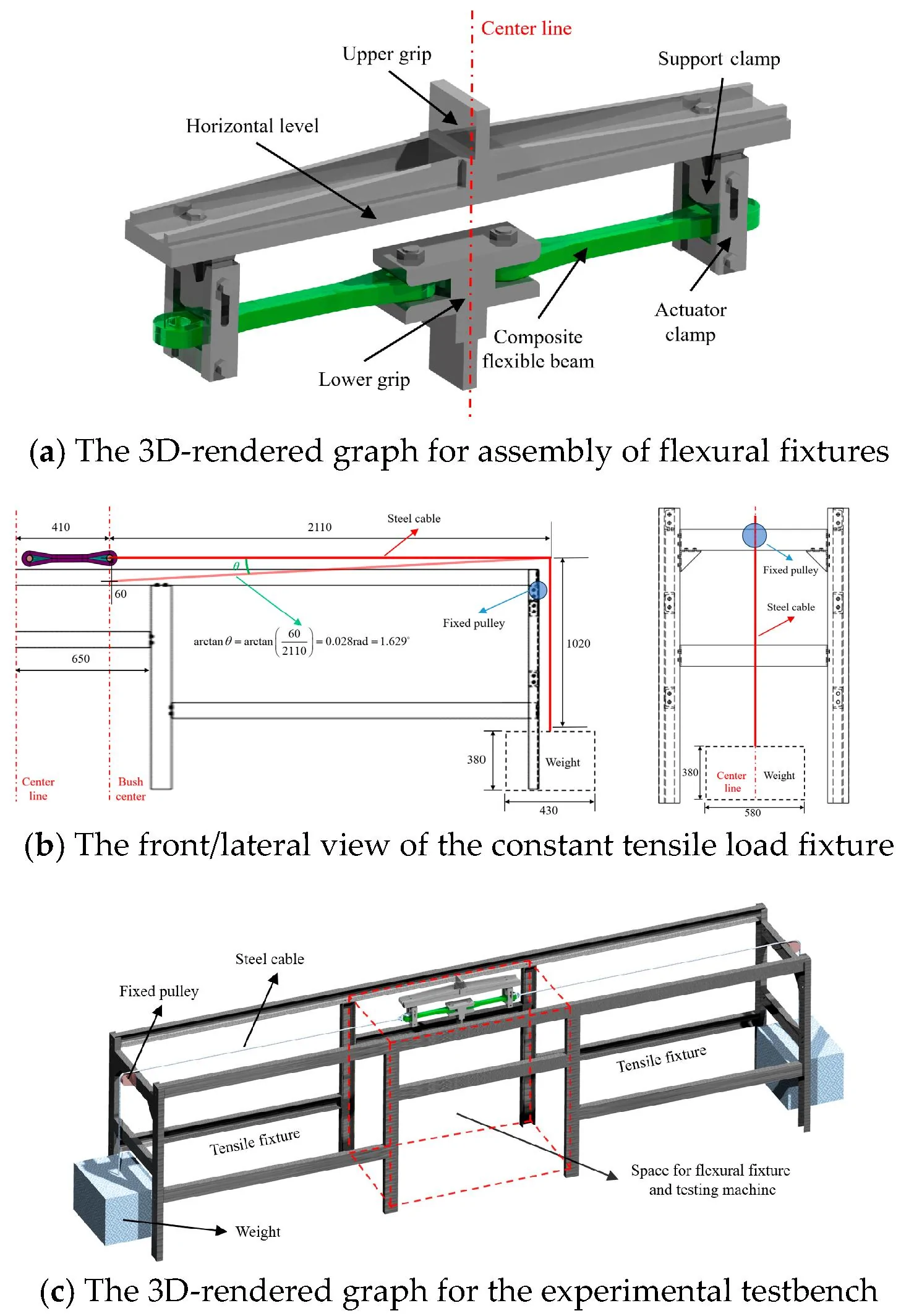
The design of the fixture and test machine.

In order to improve the experimental efficiency and more realistically simulate the working conditions of two specimens under multiaxial loads, the flexural fixture is presented in Figure 11a. The flexural fixture includes an upper grip (loading end), a horizontal level, support clamps, actuator clamps, and a lower grip (fixed end).

The constant tensile load is applied by a 5 kN weight. The metal bush of the specimen is connected to the weight by a steel cable, which passes through a fixed pulley mounted on tensile fixtures. The constant tensile load fixture is presented in Figure 11b. To stabilize the tensile load, the length of the horizontal steel cable is set as 2110 mm. Therefore, the angle *θ* reaches 1.629° when the deflection of the specimen reaches 60 mm, which ensures that the constant tensile load is stable. Accordingly, the axial component of the nominal 5 kN tensile load can be calculated as *F*_axial_ = 5kN*cos(1.629°) = 4.99798kN. Therefore, the axial tensile component remains approximately 99.96% of the nominal 5 kN load, corresponding to a maximum variation of approximately 0.04%. This variation is negligible compared with the nominal tensile load.

The experiments are carried out on a hydraulic servo fatigue testing machine WANCE HDT from Shenzhen Wance Testing Machine Co., Ltd. (504 + 254 F, ±25 kN). The strain is measured by a DH3818Y 24-channel automatic strain acquisition and processing system. Full-field deformation measurements were obtained using digital image correlation (DIC). The model of the industrial camera is MV-DK8096-30UCM, and the image acquisition frequency is 2 Hz. The corresponding experimental configuration was maintained consistently throughout the structural tests. After assembly, the experimental testbench is shown in Figure 11c.

#### 3.1.3. The Visual Inspection

**Figure 12:**
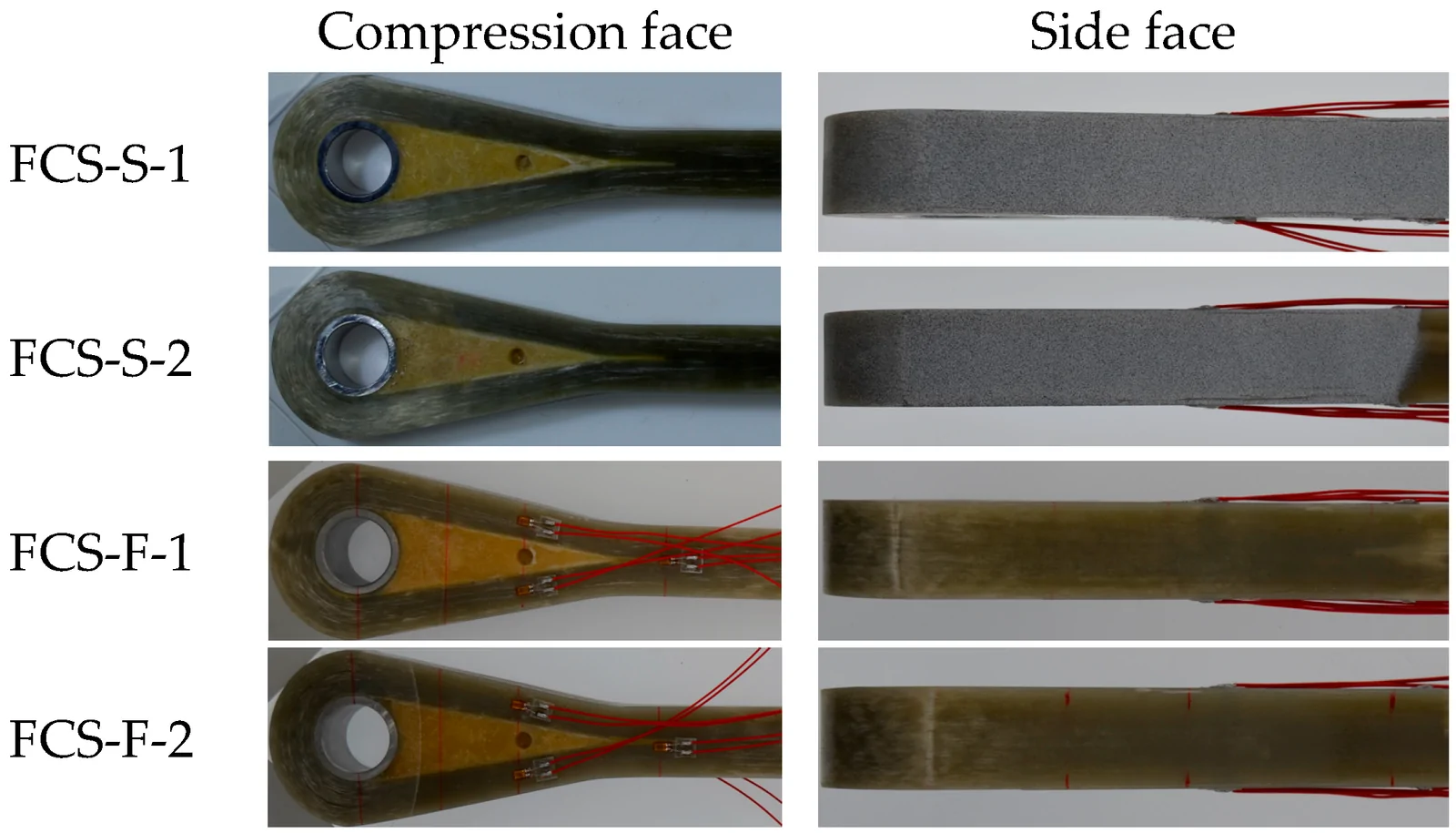
The root segments of FCSs before the experiments.

In order to compare the visual damage modes before and after experiments, it is necessary to take records of the specimen. The root segments of the specimens before the experiments are presented in Figure 12. It can be seen that there is no obvious visual damage on the surface of the specimens. In addition, it is noted that the speckles in FCS-S-1 and FCS-S-2 are in preparation for DIC measurements.

**Figure 13:**
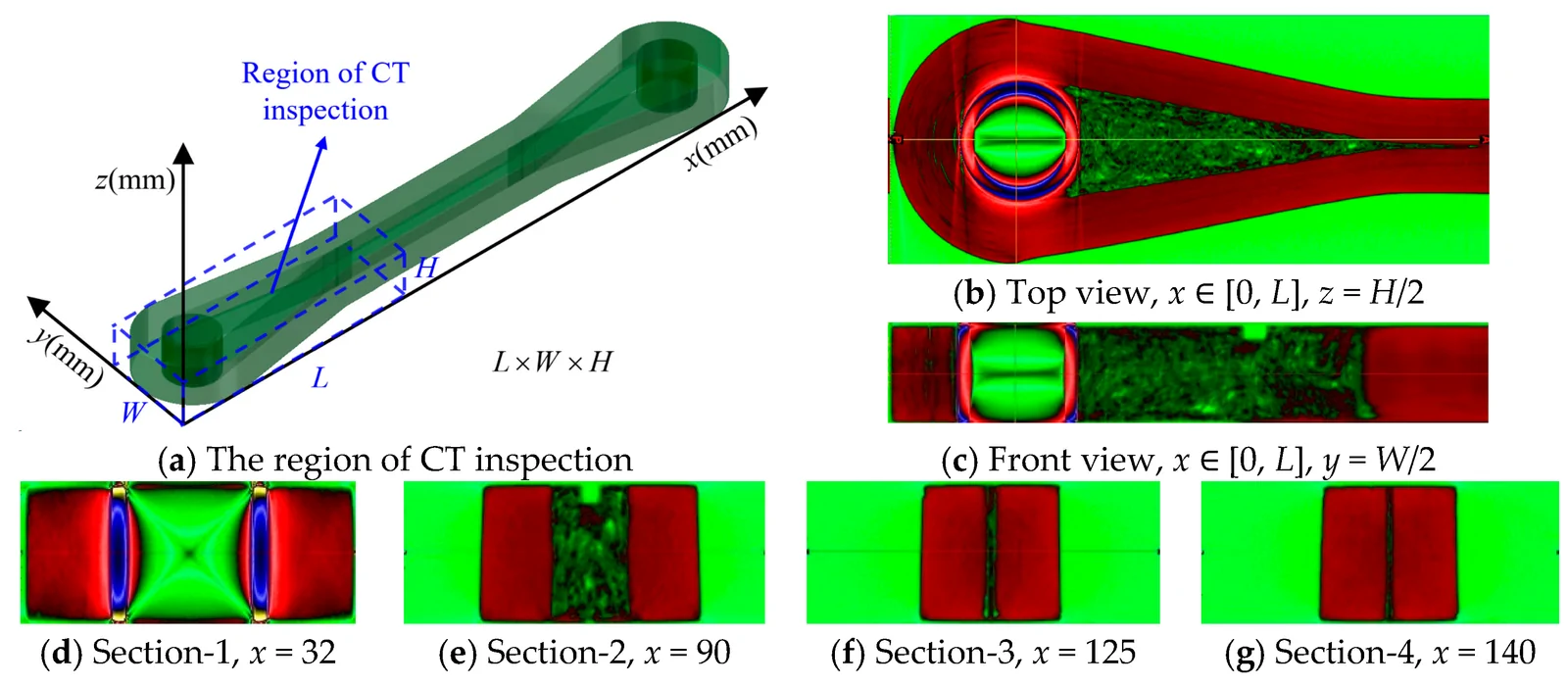
The results of CT inspection for the root segment of FCS-F-1.

Prior to mechanical testing, the internal condition of the FCS specimens was further examined using computed tomography (CT) inspection. The CT results were used to identify potential internal defects, including voids, local discontinuities, or delamination, that could not be reliably identified by external visual inspection. Representative CT images of the specimens are presented in Figure 13. The inspection results provide additional evidence of the internal condition of the specimens before mechanical testing.

### 3.2. Static Experiment

**Figure 14:**
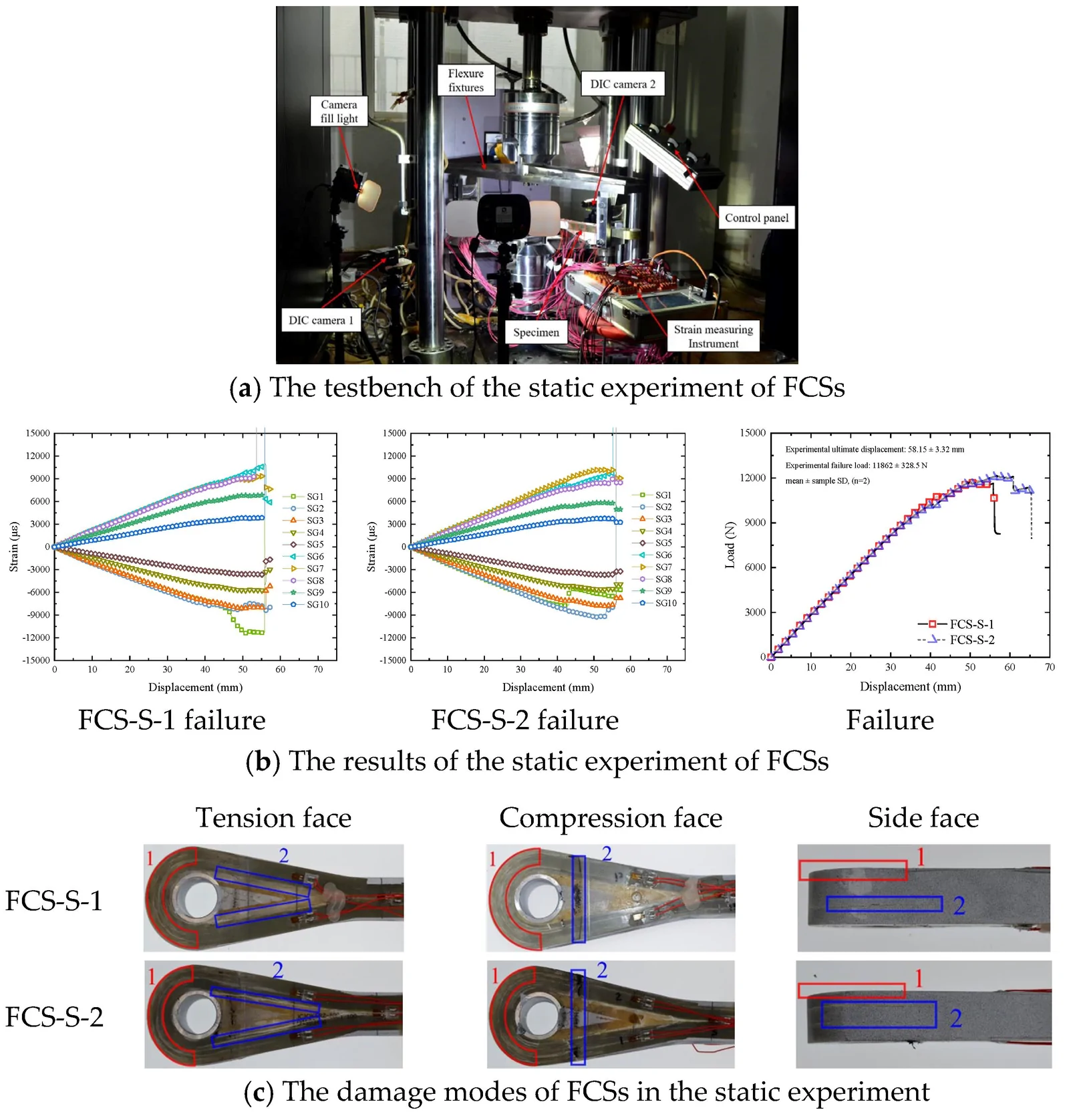
The static experiment of FCSs.

First, the static experiment of FCSs was carried out, which was demonstrated in Figure 14a. The testbench mainly includes a test machine, a flexure fixture, DIC cameras, a camera fill light, and a strain-measuring instrument.

Before the static experiment, the specimens were preloaded with 2000 N so that the symmetry of the testbench could be calibrated. Then, the strain–displacement and load–displacement curves are presented in Figure 14b. It can be found that the specimens are subjected to flexure load without obvious torsional load. Furthermore, the symmetry and consistency of the two specimens are stable, so that the reliability of the experiment can be assured. The ultimate responses of the two static FCS specimens were evaluated individually. FCS-S-1 reached an ultimate displacement of 55.8 mm and a failure load of 11,630 N, while FCS-S-2 reached an ultimate displacement of 60.5 mm and a failure load of 12,094 N. The corresponding mean ultimate displacement and failure load were 58.15 mm and 11,862 N, respectively. The relatively close ultimate responses of the two specimens indicate good experimental consistency. Therefore, FCS-S-1 was selected as the representative experimental specimen for the subsequent detailed comparison with the numerical results. The damage modes are recorded in Figure 14c to compare with those in Figure 12.

On the tension face, the matrix damage in red region 1 and the delamination damage in blue region 2 have occurred. On the compression face, the matrix damage in red region 1 is more serious than that of the tension face, and the fiber damage in blue region 2 is the main cause of FCS’s failure. On the side face, the matrix damage in red region 1 corresponds to that of the compression face, and some cracks because of matrix damage in blue region 2 have occurred.

### 3.3. Fatigue Experiment

**Figure 15:**
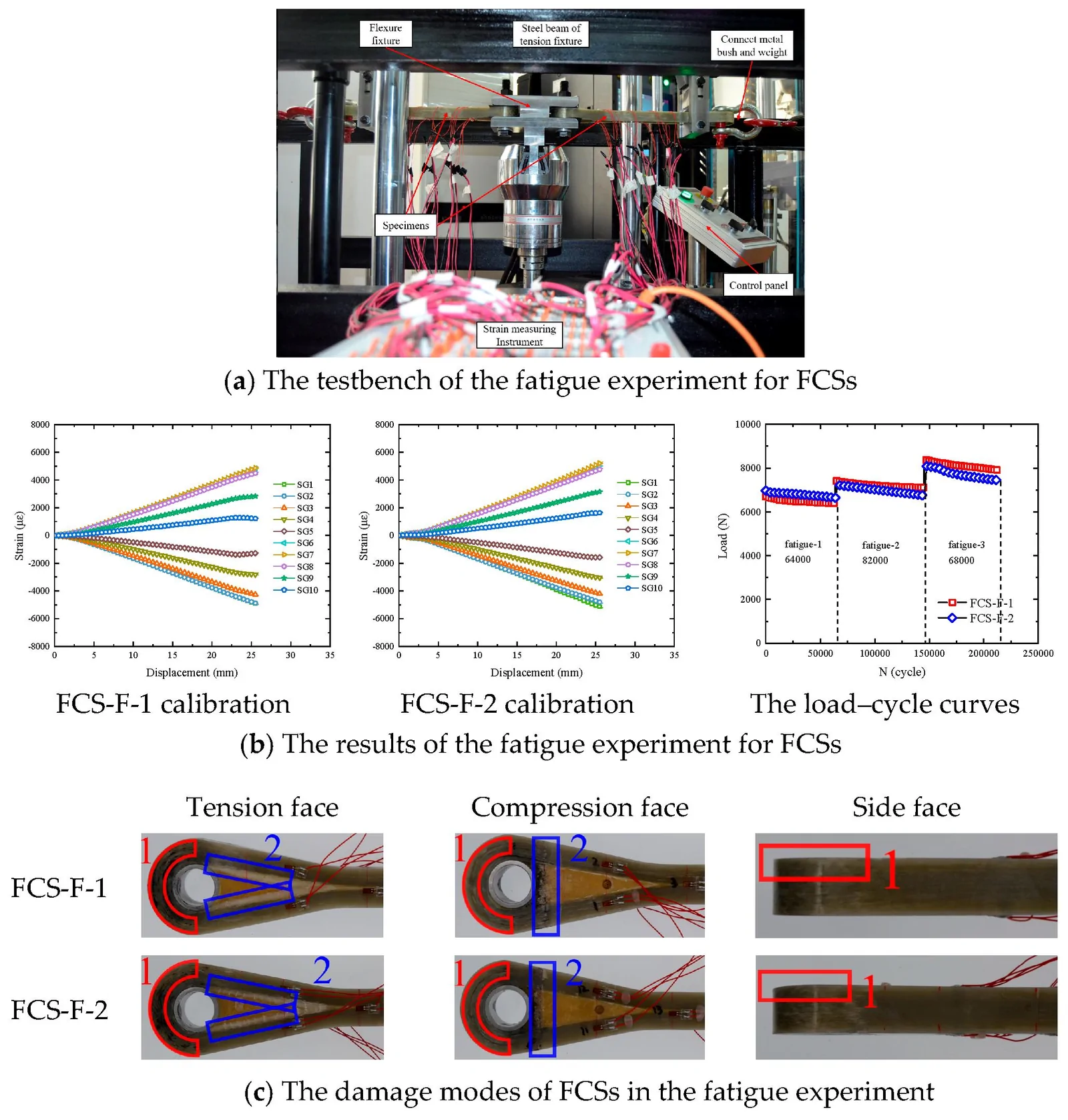
The fatigue experiment of FCSs.

Then, the fatigue experiment for FCSs is carried out, which is presented in Figure 15a. The testbench mainly includes a test machine, a flexure fixture, a tension fixture, a weight, and a strain-measuring instrument. Before the fatigue experiment, the specimens were first preloaded with 3000 N, so that the symmetry of the testbench could be calibrated. Then, the strain–displacement and load–displacement curves are presented in Figure 15b. These results demonstrate good experimental consistency for the tested specimens. However, because only two specimens were tested, this consistency should be regarded as experimental repeatability rather than a statistical assessment of structural robustness.

Then, the FCSs are subjected to a constant tensile load of 5 kN and three stages of flexural cyclic displacement (*R* = 0). The staged displacement protocol was designed to characterize the progressive degradation of FCS structural fatigue stiffness under gradually increasing fatigue stress levels. (1) The first-stage fatigue displacement was determined from the nonlinear response identified in the static test. The nonlinear displacement was 40.46 mm, and 65% of this value was selected according to the material-level fatigue loading design experience, giving an initial fatigue displacement of approximately 26.3 mm. The corresponding fatigue stress level was quantified from the average strain measured by SG-1, SG-2, SG-6, and SG-7 and normalized by the static failure strain (9244 με), resulting in a stress level of 55.2%. (2) The transition between fatigue stages was determined from the evolution of the structural fatigue load. For FCS-F-1, when the fatigue load decreased to approximately 95.30% of its initial value and tended toward a relatively stable state after 64,000 cycles, the first stage was terminated. (3) To promote further fatigue damage evolution, the target stress level was increased to approximately 60%. The corresponding fatigue strain was calculated as 5546 με, and displacement-controlled static loading gave a fatigue displacement of 27.5 mm. After the loading/unloading process, the measured fatigue strain was 5655 με, corresponding to an actual stress level of 61.2%. The second stage was terminated when the fatigue load decreased to approximately 90.43% of its initial value after 82,000 cycles. (4) The third stage was subsequently conducted at a fatigue displacement of 29.8 mm, corresponding to a stress level of 68.3%, and was terminated after 68,000 cycles when the fatigue load decreased to approximately 85.05% of its initial value. (5) The same three-stage displacement history was subsequently applied to FCS-F-2 to examine the repeatability of the structural fatigue stiffness degradation.

The load–cycle curve for the three stages is presented in Figure 15b. The three stages of fatigue cycles of FCS-F-1 are summarized in Table 8. In the present displacement-controlled structural fatigue test, the fatigue stress level was quantified using the measured strain ratio, defined as the ratio of the average strain measured by the selected strain gauges under fatigue loading to the corresponding static failure strain. It should be noted that this strain ratio is used as an experimentally measurable stress-level indicator and is not assumed to be strictly equivalent to the true stress ratio under multiaxial loading and progressive structural damage.

The three fatigue stages consisted of 64,000, 82,000, and 68,000 cycles, respectively, resulting in a total of 214,000 cycles for each fatigue specimen. Thus, the reported total cycle count corresponds to the prescribed representative test history applied to each specimen rather than an average fatigue life obtained from multiple specimens. It should also be noted that the present structural fatigue test does not define a conventional final-failure cycle. Instead, the test is intended to characterize the progressive reduction in structural fatigue stiffness under the prescribed displacement history. The termination of each stage was determined by the stabilization of the measured fatigue load at the corresponding displacement level. The two fatigue specimens exhibited consistent fatigue-load degradation under the same three-stage loading history.

Because the fatigue test was conducted under displacement control, the structural fatigue stiffness was calculated as the ratio of the peak load to the corresponding peak displacement: *K*_FCS_ = *F*_peak_/*δ*_peak_, where *F*_peak_ and *δ*_peak_ denote the peak load and peak displacement of the corresponding fatigue cycle, respectively. Within each prescribed displacement stage, the peak displacement remained essentially constant, while the peak load decreased with progressive fatigue damage. Therefore, the reduction in *K*_FCS_ represents the progressive degradation of the structural fatigue stiffness.

The fatigue damage modes are presented In Figure 15c. On the tension face, the matrix damage in the red region 1 is relatively slight and barely visible, whereas signs of delamination can be observed in the blue region 2. On the compression face, the matrix damage in the red region 1 is more pronounced than that on the tension face, while fiber damage in the blue region 2 remains the dominant damage mode associated with the fatigue failure and fatigue stiffness degradation of the FCS. On the side face, the matrix damage in the red region 1 is similar to that observed on the compression face, accompanied by slight crushing and local deformation. The two fatigue specimens exhibited consistent damage locations and failure characteristics under the same three-stage displacement history, supporting the repeatability of the observed structural fatigue damage evolution.

In conclusion, four FCS specimens were tested in total, including two specimens for static testing and two specimens for fatigue testing. The individual experimental results are presented in Section 3.2 and Section 3.3, and the two static specimens and two fatigue specimens exhibited consistent responses and failure characteristics. Nevertheless, the limited number of specimens is insufficient for a statistical characterization of structural fatigue variability or robustness. The small sample size was primarily due to the high manufacturing and testing cost of the FCS specimens. Therefore, the structural experiments are regarded as independent validation cases for the proposed fatigue model and FPDM rather than as a statistically sufficient database for structural reliability assessment.

## 4. Validation

### 4.1. Finite Element Models (FEMs)

The two static FCS specimens were not assigned separately to model calibration and independent validation. Instead, both specimens were tested independently to assess the repeatability of the structural response. The 3D-FEM was established based on the experimentally characterized material properties and structural information, rather than by independently fitting the numerical response to one of the two FCS specimens. Because the two specimens exhibited consistent experimental responses, FCS-S-1 was selected as the representative experimental case for the detailed model-to-test comparison presented below. The second specimen provides independent evidence of the repeatability of the structural experiment.

Considering the symmetry of the model, only one FCS is modeled. In order to apply accurate boundary conditions (BCs), the modeling of the lower grip in Figure 11a is carried out. The FCS was modeled using C3D8R solid elements with three-dimensional orthotropic material properties. The in-plane elastic constants and strength parameters of 3232A/Glass are listed in Table 1 and were experimentally characterized in the previous study [39]. For the out-of-plane properties required by the three-dimensional solid-element formulation, transverse-isotropy assumptions were adopted, namely E_3_ = E_2_, G_13_ = G_12_, and v_13_ = v_12_. The remaining parameters were obtained from the manufacturer’s material data, with v_23_ = 0.41 and G_23_ = 3.5 Gpa. These parameters were used to complete the three-dimensional material definition of the C3D8R elements.

**Figure 16:**
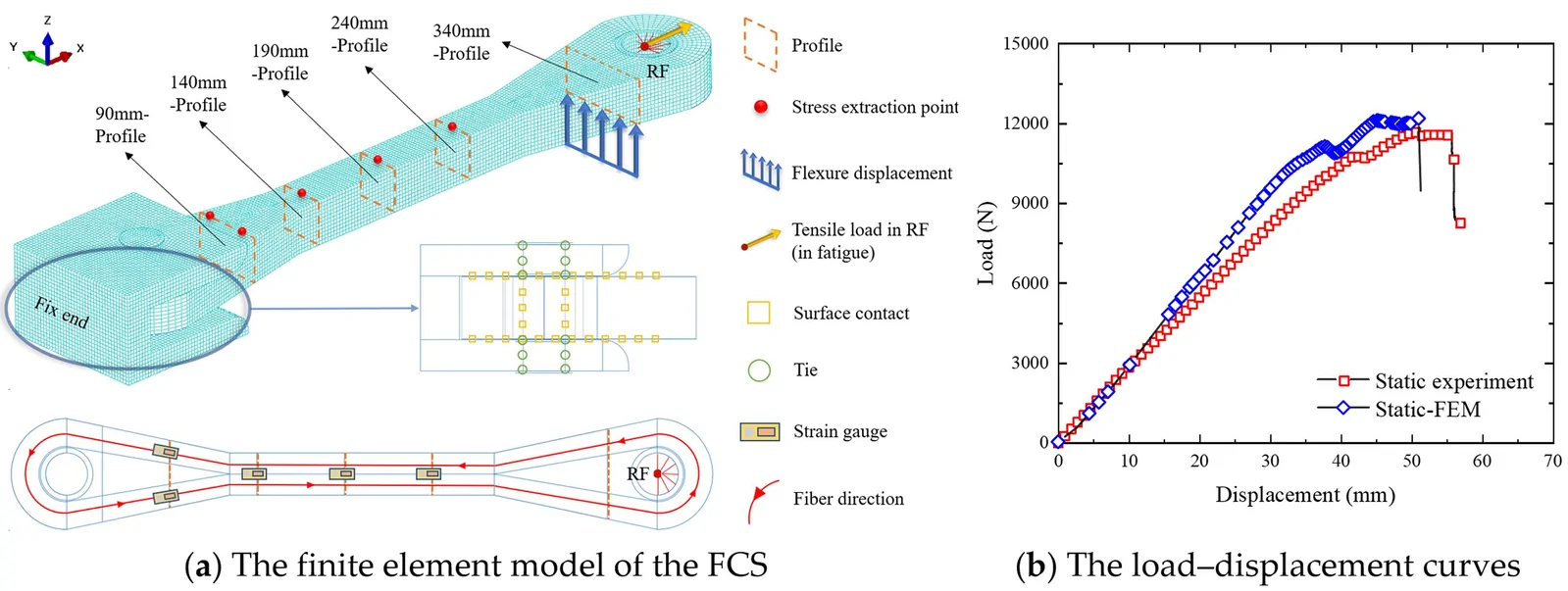
The 3D-FEM of the FCS in static experiments.

A mesh-convergence analysis was performed to determine an appropriate global mesh size for the FCS 3D-FEM. Four global mesh sizes of 10, 5, 2, and 1 mm were investigated. The corresponding structural stiffnesses were 350.56, 328.19, 318.14, and 315.84 N/mm, respectively. The results show that the variation in structural stiffness becomes relatively small when the mesh size is reduced from 2 to 1 mm. Considering both numerical convergence and computational efficiency, a global mesh size of 2 mm was selected for the subsequent simulations. The number of C3D8R elements is 44,760. The details of sections, interactions, BCs, and material direction are presented in Figure 16a.

The boundary conditions and load application used in the 3D-FEM are illustrated in Figure 16. The fixture was fully constrained. Surface-to-surface contact was defined between the inner surfaces of the fixture and the upper and lower surfaces of the FCS root. The contact surfaces between the clamping bolts, fixture, and metal bush were defined using tie constraints. A displacement load in the positive z-direction was applied at the 340 mm profile of the FCS. A reference point was defined at the center of the metal bush on the side opposite to the loading end, and the reference point was coupled to the inner surface of the bush. A constant centrifugal load along the longitudinal direction of the FCS was then applied directly to this reference point.

The winding prepreg, filling block, and metal bush were constructed as a single geometric part and partitioned into different material regions. Therefore, the interfaces between these regions were modeled as perfectly bonded interfaces, equivalent to a tie constraint, and no cohesive-zone or interface-debonding behavior was introduced. This assumption was adopted because reliable fatigue debonding parameters for the relevant interfaces were not available. In addition, post-test nondestructive inspection did not indicate newly developed debonding between the winding prepreg and the filling block or metal bush. The bonded regions were also designed to have higher strength than the interlaminar strength of the composite material. Nevertheless, interfacial fatigue delamination/debonding may affect the structural fatigue response and represents an important topic for future investigation.

Because the FCS is subjected to a complex bending and multiaxial stress state, the experimentally measured strain was also compared directly with the FEM-predicted strain at the corresponding strain-gauge locations. This direct comparison avoids introducing an additional assumption of a locally uniaxial constitutive state through *S*_11_ = *E*_1_*ε*. The FEM-predicted strain distributions at the corresponding gauge locations show good agreement with the experimental measurements. Therefore, the numerical model is evaluated using both the stress response and the directly measured strain response. Comparison of the FEM-predicted and experimentally measured responses at the strain-gauge locations is summarized in Table 9.

After that, the static analysis of the FCS is carried out. The failure criterion is given in Table 2, and the material properties are suddenly reduced based on the failure modes. Finally, the load–displacement curves are presented in Figure 16b. The failure displacement obtained by the FEM is 51.3 mm, which deviates from the experimental data (55.8 mm) by 7.99%. The ultimate failure load obtained by the static-FEM is 12,180 N (6090 N × 2), which deviates from the experimental data (11,630 N) by 4.73%. Beyond the ultimate load and displacement, the initial stiffness and onset of nonlinearity were also compared between the experiment and the 3D-FEM. The initial stiffness obtained from the static experiment was 291.72 N/mm, while that predicted by the 3D-FEM was 318.14 N/mm. Taking 95% of the initial stiffness as the criterion for the onset of nonlinear structural response, the corresponding displacement was 40.46 mm in the experiment and 37.68 mm in the numerical simulation. The damage-initiation sequence was also consistent between the experiment and simulation.

**Figure 17:**
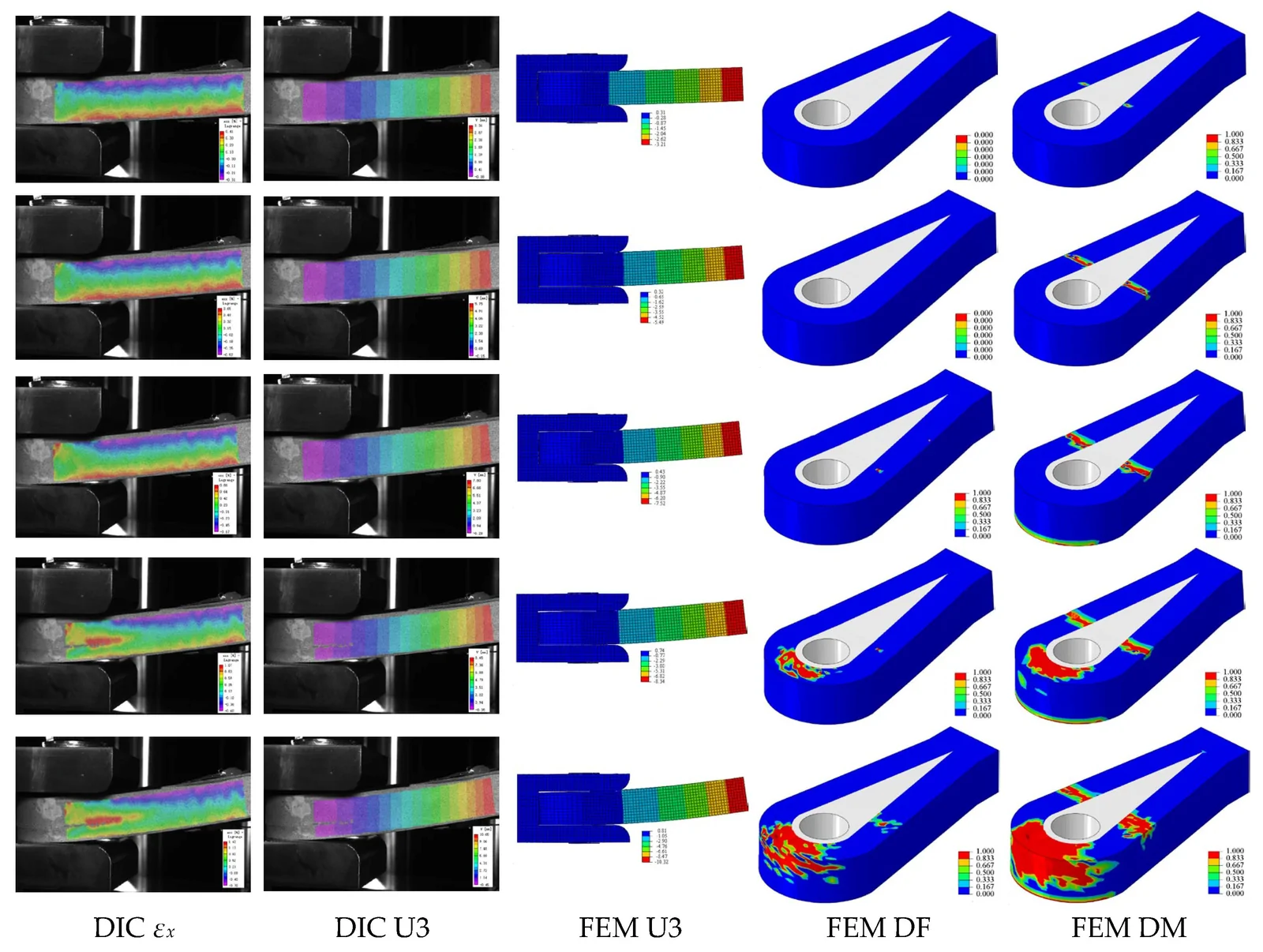
The progressive damage process of the FCS by DIC and static-FEM.

To further quantify the agreement between the DIC measurements and the FEM results, the DIC and FEM loading histories were aligned according to the corresponding loading time, as illustrated in Figure 17. The displacement field in the U3 direction obtained from DIC shows the same overall evolution as that predicted by the FEM, and the maximum displacement discrepancy throughout the static test was approximately 4.5%. In addition, the experimentally measured ε_x_ field was compared with the FEM damage variables. At the compressed root region of the FCS, the strain concentration observed in the DIC results coincides with the initiation of the corresponding FEM damage. With increasing load, the redistribution of the DIC ε_x_ field is consistent with the progressive expansion of the fiber-damage region predicted by the FEM. These results indicate that the FEM reproduces not only the global displacement response but also the location and evolution of the dominant damage region observed experimentally. It should be noted that DIC provides deformation and strain fields rather than direct identification of the physical damage mechanism; therefore, the DIC results are used here to correlate deformation localization with the FEM-predicted damage evolution.

### 4.2. Fatigue Analysis

**Figure 18:**
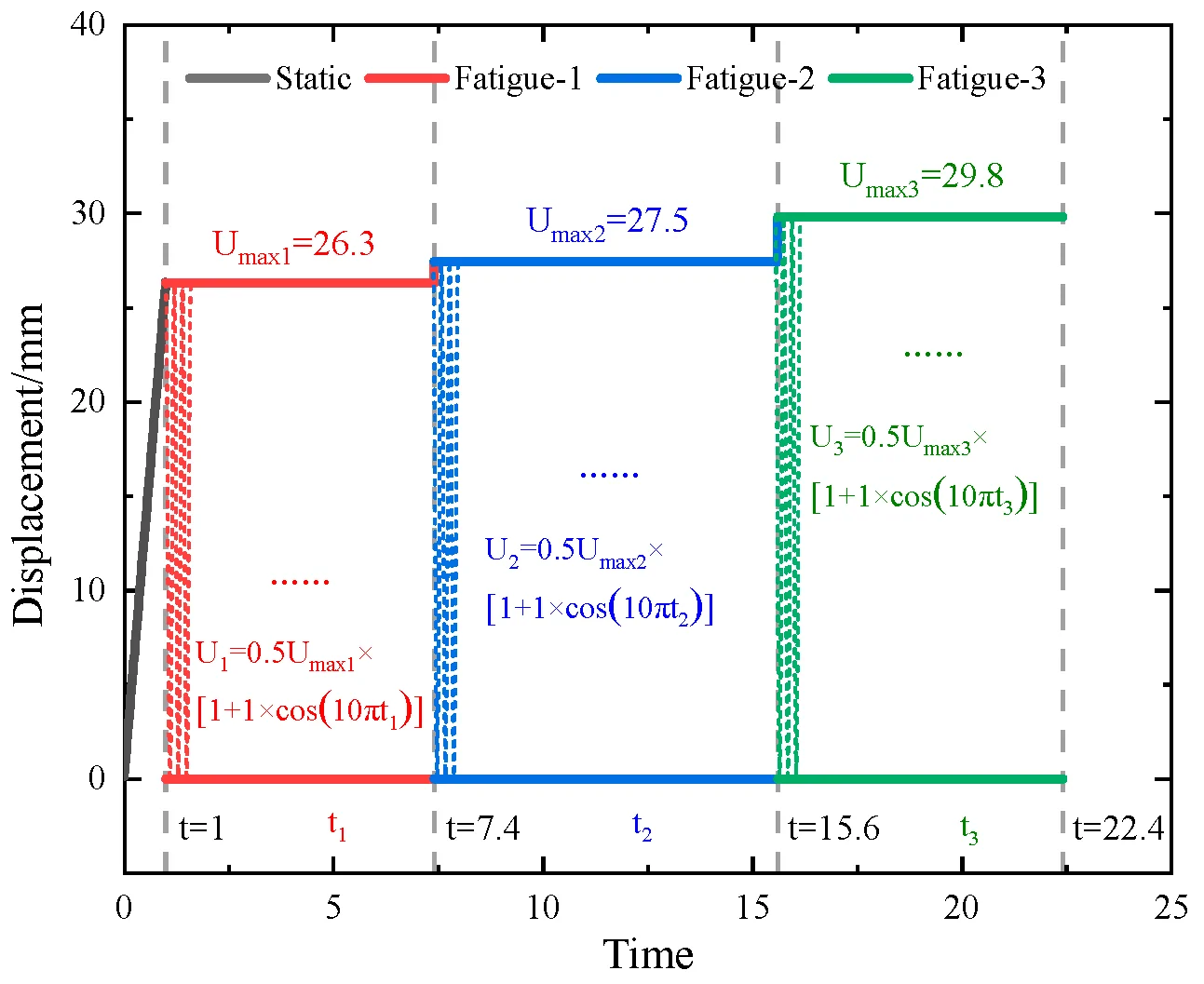
The flexural cyclic displacements of the fatigue-FEM analysis.

The FEM and BCs of the fatigue analysis are exactly the same as those of the static analysis. It should be noted that a 5 kN tensile load is applied to the reference point (RF), which is presented in Figure 16a. In Table 8, the flexural cyclic displacements at the three stages are described by a cosine function. The complete analysis is demonstrated in Figure 18, which includes static, fatigue-1 (64,000), fatigue-2 (82,000), and fatigue-3 (68,000). The frequency of flexural cyclic displacements in the FEM analysis is 5 Hz. It should be noted that the 5 Hz frequency used in the fatigue FEM does not represent the physical fatigue frequency of the experiment, which was 0.5 Hz. In the numerical analysis, the loading frequency is used only as a computational parameter for applying the prescribed cyclic displacement history. The fatigue constitutive model is cycle-based, and the evolution of fatigue damage is updated according to the accumulated fatigue cycle number rather than the physical elapsed time. Therefore, the numerical loading frequency and the experimental loading frequency have different meanings.

To accelerate the fatigue process and evaluate the influence of the cycle-jump size on the fatigue FEM results, a sensitivity analysis was performed using Δ*N* = 1000, 2000, 3000, and 10,000 cycles. A constant fatigue displacement of 35 mm and a total fatigue cycle number of 100,000 were used in all cases. The sensitivity of structural fatigue-load degradation to the cycle-jump size is shown in Table 10. The results show that the fatigue-load degradation is already approaching convergence at Δ*N* = 2000, whereas larger cycle jumps lead to an increasingly noticeable deviation. Considering both numerical accuracy and computational efficiency, Δ*N* = 2000 was selected for the subsequent fatigue FEM simulations.

**Figure 19:**
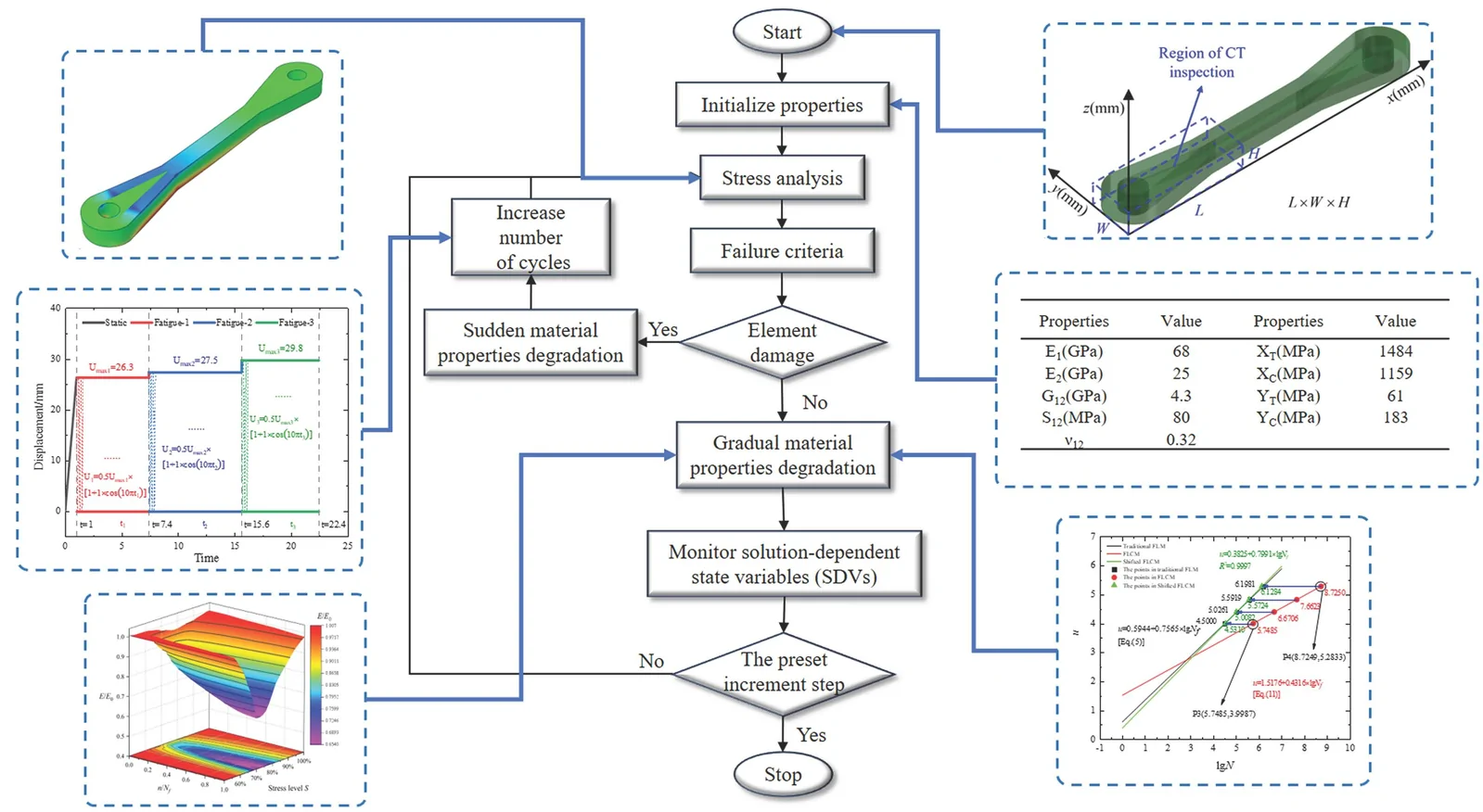
The flow chart of the FPDM in the UMAT subroutine.

The overall computational procedure of the proposed FPDM is illustrated in Figure 19. At each constitutive update, the current strain increment and the previous state variables are first used to update the local stress state. The current stress state is then provided to the fatigue-life model to determine the corresponding fatigue life and normalized fatigue state. The ANN-based fatigue stiffness degradation model is subsequently queried to obtain the instantaneous normalized stiffness (*E*/*E*_0_)_ANN_. The historical minimum stiffness condition is then enforced according to the irreversible damage rule defined in Section 2. The updated constitutive stiffness is used to calculate the current stress response, while the Hashin failure criteria are simultaneously evaluated to identify element-level damage or failure. The updated stiffness, fatigue state, damage state, and historical minimum *E*/*E*_0_ are stored as solution-dependent state variables (SDVs) and transferred to the next constitutive increment. Once local element damage or failure occurs, the associated stiffness reduction changes the local load-carrying capability and consequently causes stress redistribution to neighboring elements. The above procedure is repeated throughout the fatigue analysis.

**Figure 20:**
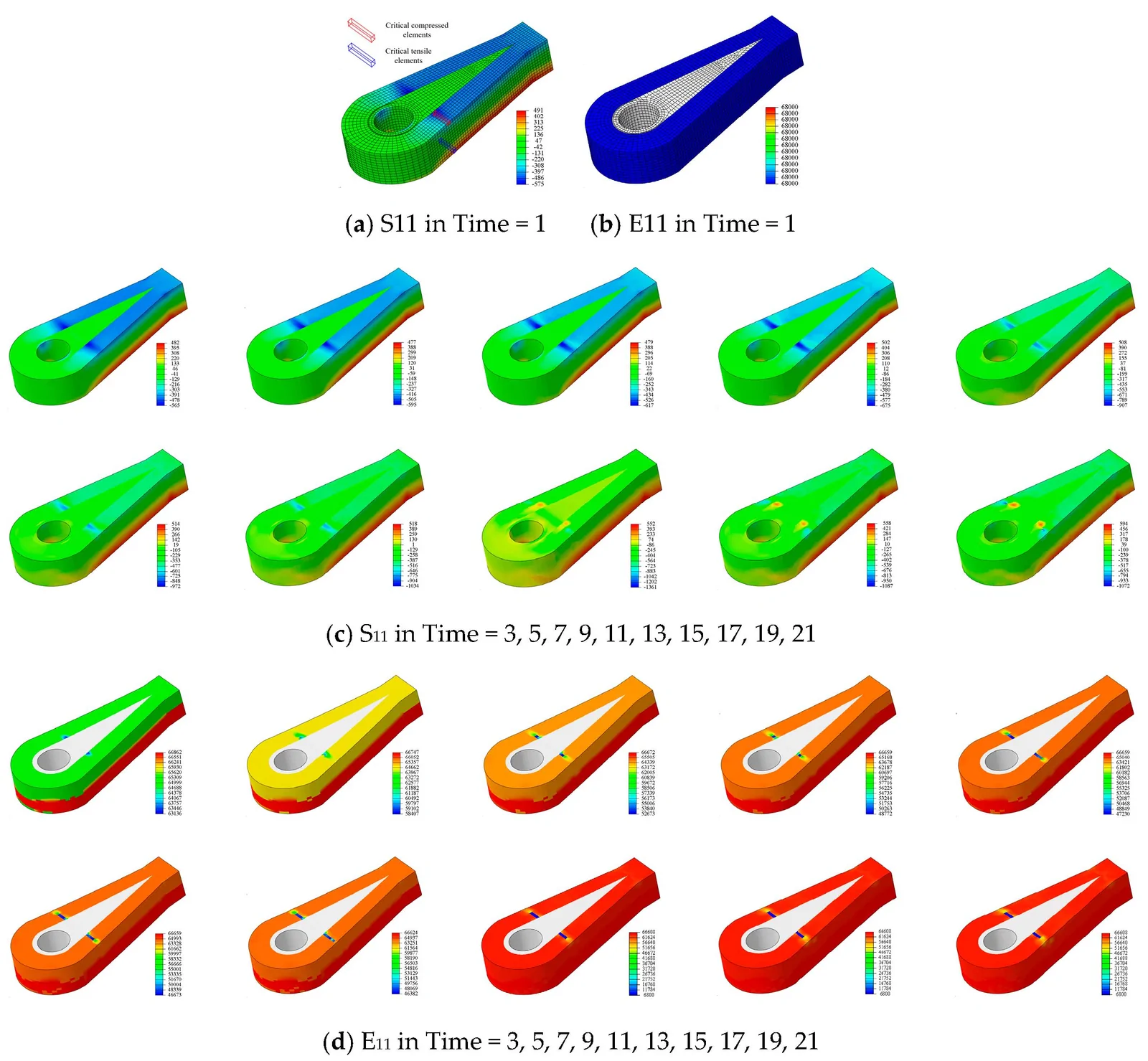
The S_11_ and E_11_ fields for the FCS in fatigue-FEM.

To illustrate the effect of fatigue stiffness degradation on stress redistribution, the stress field S_11_ and the elastic modulus field E_11_ of the root segment in the FCS are presented in Figure 20. The ‘Time = 1’ corresponds to the start of the ‘fatigue-1’ step. The ‘Time = 3, 5, 7, 9, 11, 13, 15, 17, 19, 21’ correspond to ‘fatigue cycles *N* = 20 k, 40 k, 60 k, 80 k, 100 k, 120 k, 140 k, 160 k, 180 k, 200 k’. The following conclusions can be drawn from the observations: (1) The fatigue stiffness degradation of the structure is dominated by the compressive properties in the fiber direction; (2) The stress concentration at the fixed end of the structure is the main cause of fatigue damage; (3) The tendency of stress distribution and the minimum E_11_ change significantly between *N* = 140 k and *N* = 160 k.

**Figure 21:**
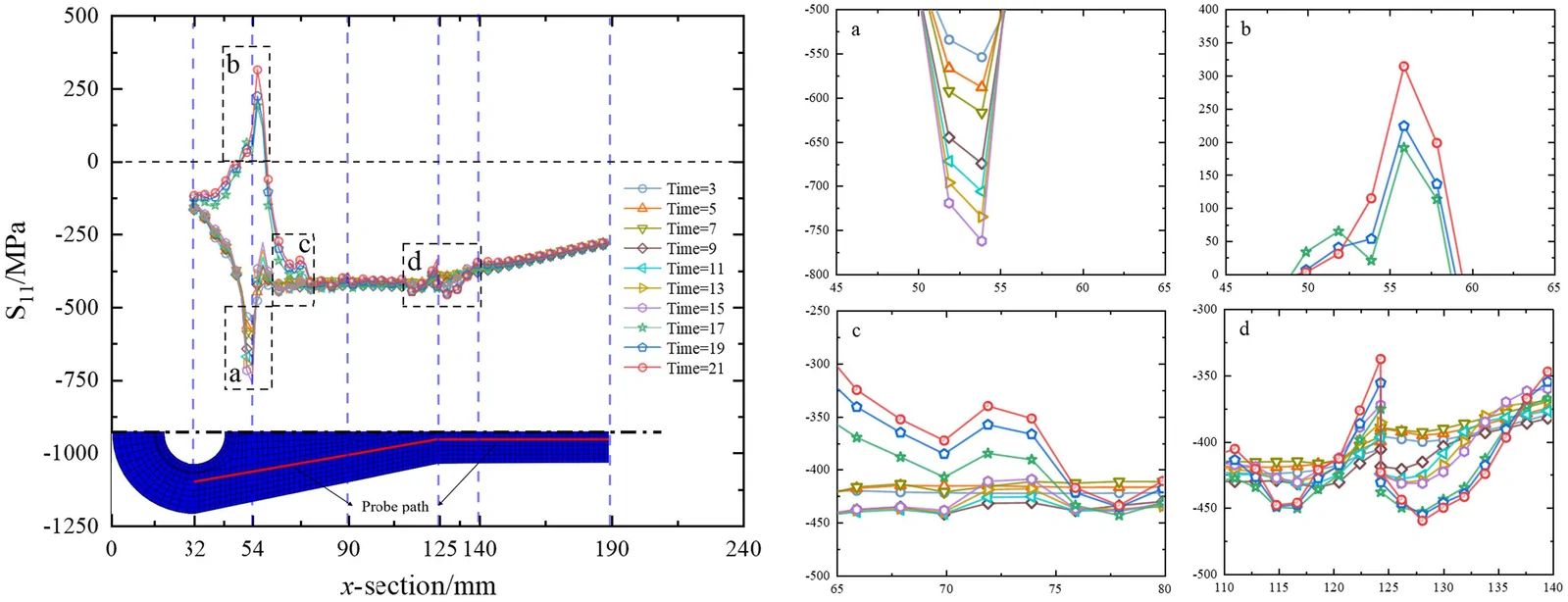
The average S_11_ distribution along the probed path in fatigue-FEM. (**a**–**d**) Local magnified diagram.

In order to display stress distributions along the FCS, a probed path is selected to research the stress distribution of elements during the fatigue process. The average S_11_ distribution and local critical sections are demonstrated in Figure 21. As the fatigue cycle increases, it can be found from the four partial pictures that: (a) The S_11_ of compressed elements around *x* = 54 (the fixed end) increases from *N* = 0 to *N* = 140 k; (b) The elements at *x* = 54 fail between *N* = 140 k and *N* = 160 k, which causes the stress state of elements around *x* = 54 to change from compression to tension. The fatigue damage causes the stress distribution (*x* = 54–125 mm) of the FCS to change from stable to steep; (c) The change of stress distribution indicates that the fatigue damage propagates from the compression surface at *x* = 54 to the thickness and length directions of the structure; (d) The S_11_ of compressed elements around *x* = 125 (the end of the filling block) fluctuates greatly. It can be predicted that the bonding between the winding prepreg and the end of the filling block is prone to failure under high-cycle flexure fatigue.

The stress redistribution observed around *x* = 54 mm and *x* = 125 mm is obtained primarily from the numerical evolution of the FPDM. Direct experimental strain/DIC measurements at these locations were not available throughout the fatigue history. In particular, the *x* = 54 mm region is located within the clamping region, which prevents the installation of surface-mounted strain gauges in the same manner as in the free structural region. Therefore, the local stress-redistribution results should be regarded as a numerical interpretation supported by the experimentally observed global fatigue stiffness degradation and final damage characteristics, rather than as a directly measured local experimental quantity.

**Figure 22:**
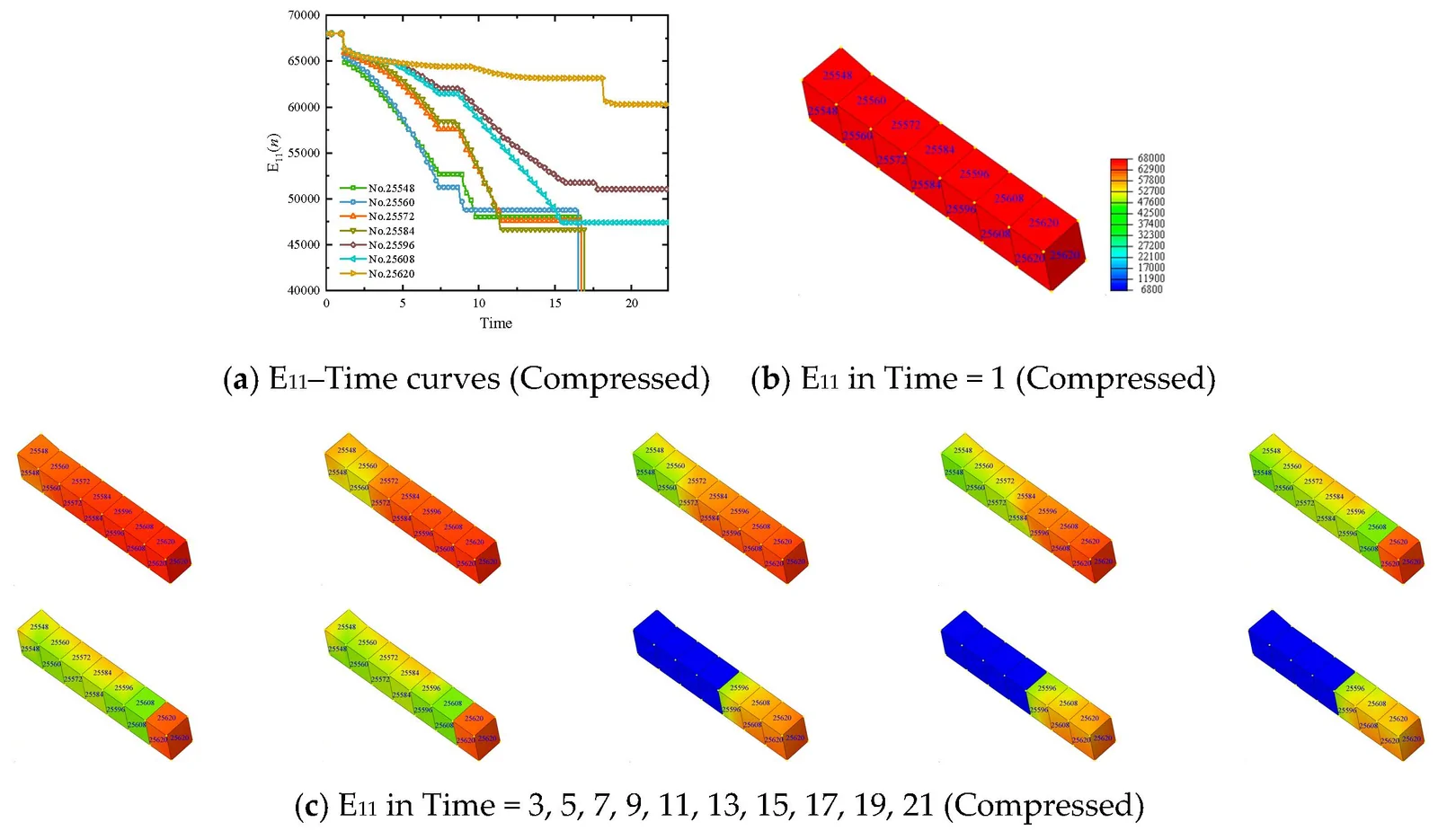

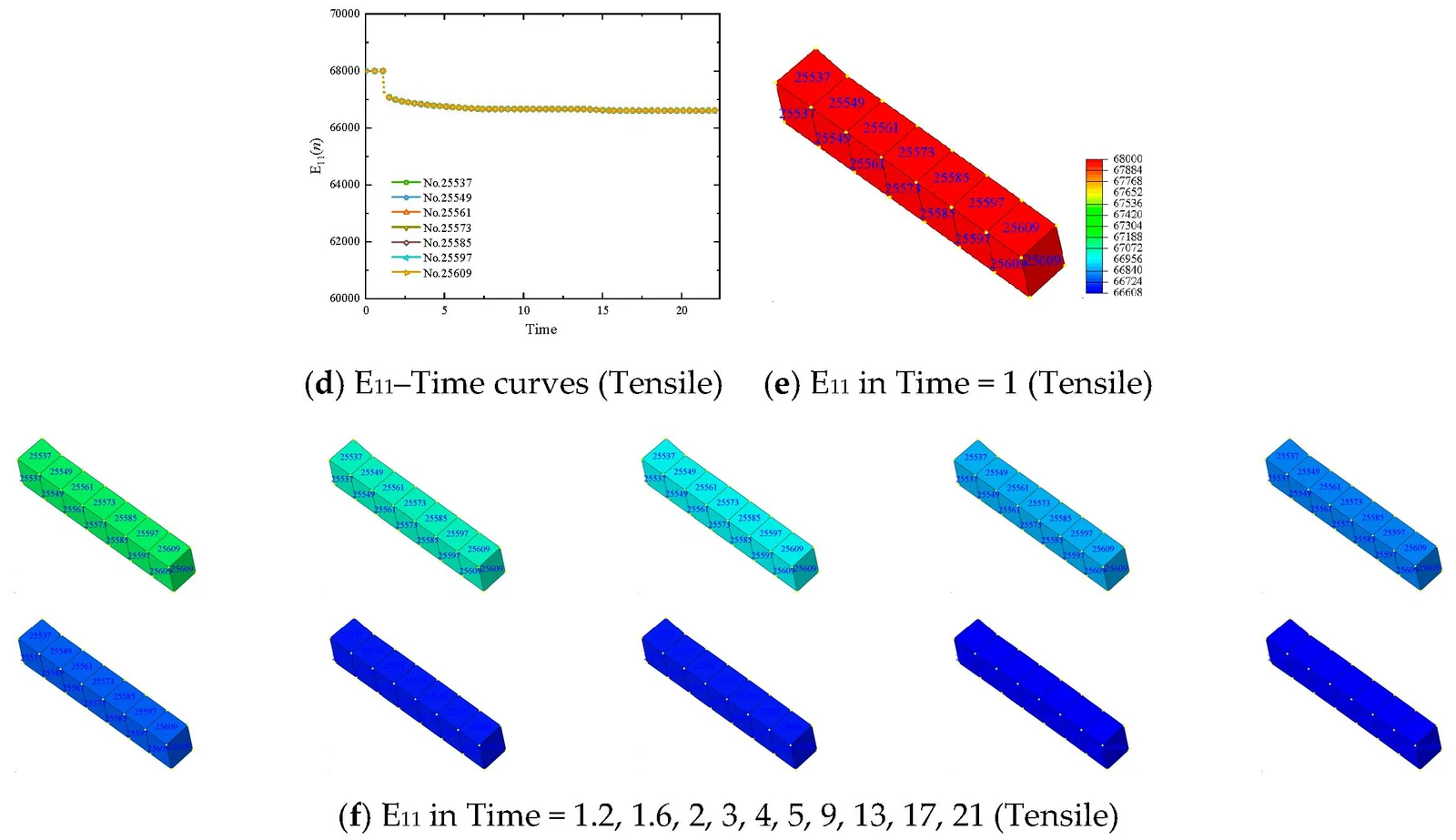
The E_11_ of critical elements in fatigue-FEM.

**Figure 23:**
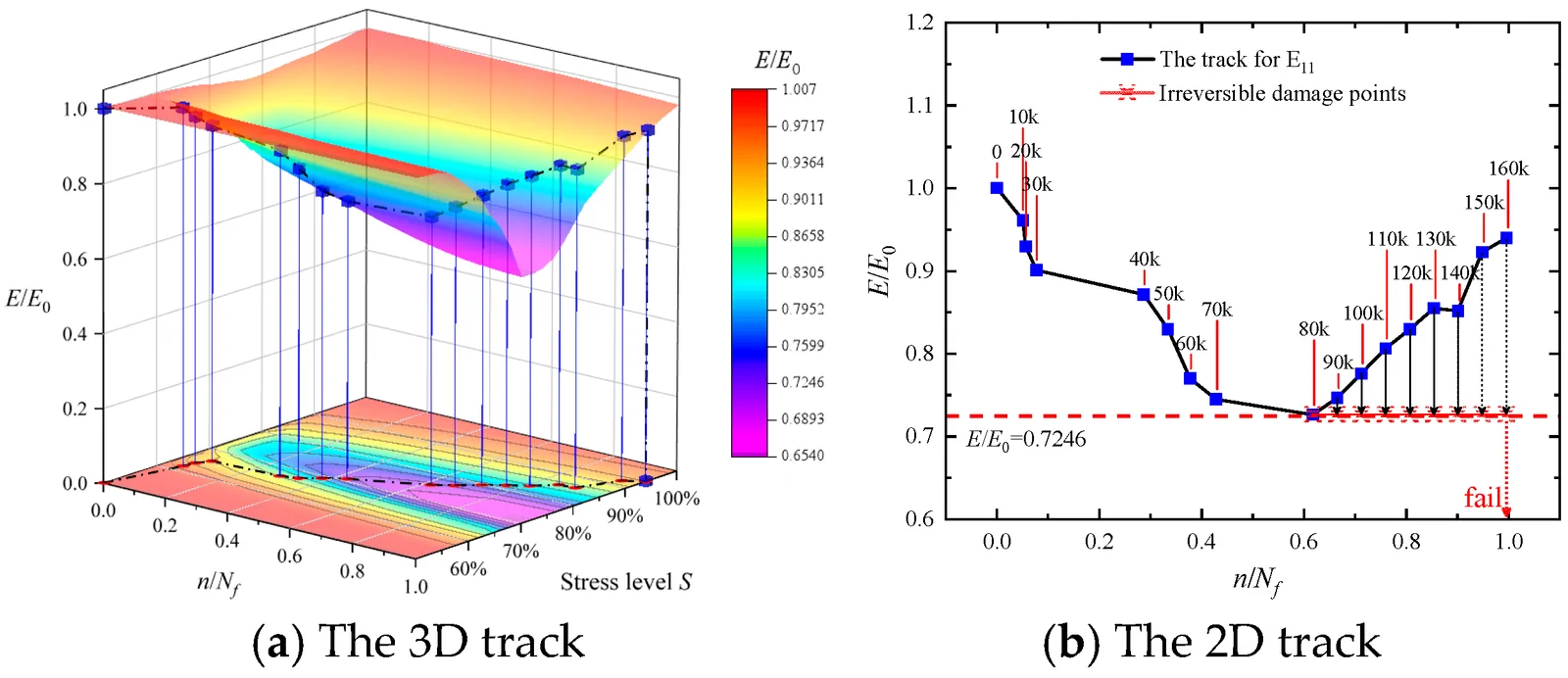
The trajectory for E_11_ of element No.25560 in the response surface of 0° C–C fatigue.

In order to display the fatigue degradation at the fixed end of the FCS directly, seven critical elements on the compression and tension surfaces in the root segment are selected, respectively (labeled in Figure 20a). The E_11_ of critical elements during the fatigue process are demonstrated in Figure 22. Several observations can be summarized: (1) The E_11_ of compressed elements drops more suddenly than that of tensile elements at *N* = 0; (2) The degradation rates of E_11_ in compressed elements have an obvious boundary effect, while the degradation rates of E_11_ in tensile elements are uniform; (3) Because the damage is assumed to be irreversible, it could have a plateau for E_11_ in the process of fatigue degradation, which will be clearly illustrated in Figure 23; (4) The compressed elements No.25548, No.25560, No.25572, and No.25584 fail at *N* = 158 k, 156 k, 158 k, and 160 k, respectively.

The predicted failure of the compressed elements occurs at approximately 156–160 k cycles. This element-level failure is a model-internal event and should not be interpreted as a directly measured experimental failure cycle. Nevertheless, it is closely associated with the subsequent acceleration of structural stiffness degradation predicted by the fatigue FEM. The corresponding experimental fatigue-load curve exhibits a pronounced change in degradation behavior in the same stage of the fatigue history. Therefore, the predicted compressed-element failure is interpreted as the numerical origin of the accelerated structural stiffness degradation rather than as a directly observable individual element-failure event.

In order to present the advantages of the proposed FPDM based on the FLCM and FSDM-ANN, the trajectory for E_11_ of compressed element No.25560 in the response surface of 0° C–C fatigue (presented in Figure 10b) is shown in Figure 23a,b. Each track point is separated by 10 k fatigue cycles. It can be clearly found from Figure 23a that *E*/*E*_0_ of element No.25560 degrades along with the response surface according to the calculated stress level and normalized fatigue life. Actually, based on the RFML developed for calculating the FLCM and the FSDM-ANN developed for considering a wide range of stress levels, the proposed FPDM makes sure that the material properties of integration points can follow the smooth and accurate response surface during the fatigue damage process. In Figure 23b, the projection of the track points on the *xz*-plane is given and the corresponding fatigue cycles for track points are labeled. It should be noted that the *E*/*E*_0_ increases after 80 k cycles, which violates the assumption of irreversible fatigue damage. Thus, the original red symbols are replaced with the modified blue symbols, which maintain the historical minimum of *E*/*E*_0_ = 0.7246 until failure.

**Figure 24:**
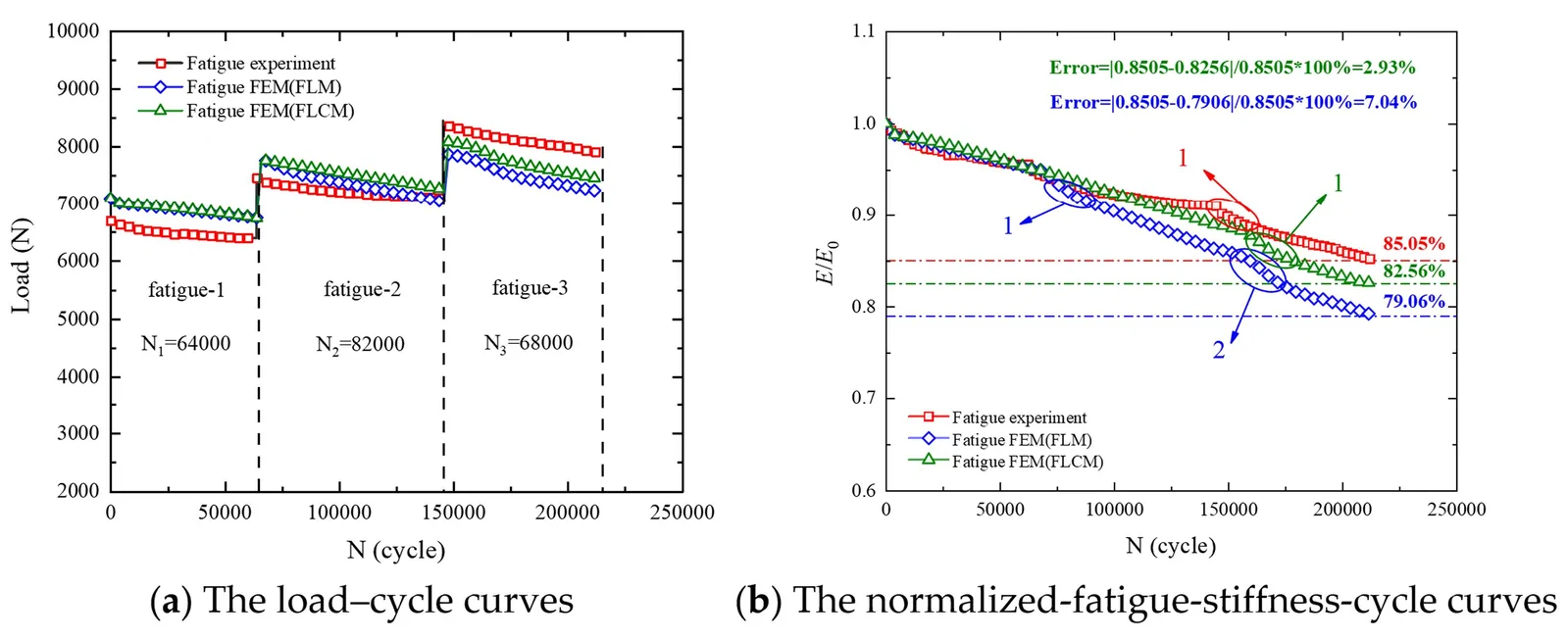
The comparison between fatigue-FEMs and the fatigue experiment of the FCS.

Finally, in order to present the improvement of the proposed FLCM relative to the traditional FLM, the curves of load–cycle and normalized-fatigue-stiffness-cycle in three stages (fatigue-1, fatigue-2, and fatigue-3) are shown in Figure 24.

Because the three fatigue stages were applied sequentially, the normalized stiffness at the end of each stage represents the accumulated stiffness degradation from all preceding fatigue cycles. The initial stiffness of each subsequent stage was therefore not reset to the undamaged stiffness; instead, the degradation continued from the normalized stiffness reached at the end of the preceding stage. Consequently, the normalized stiffness values at 64,000, 146,000, and 214,000 cycles provide stage-wise representations of the complete cumulative fatigue stiffness degradation history.

To further evaluate the fatigue FEM results over the complete three-stage fatigue history, the normalized structural fatigue stiffness at the end of each fatigue stage was compared among the experiment, the traditional FLM, and the revised fatigue-life model. The experimental result was taken as the reference value for calculating the relative error. The results are summarized in Table 11. The stage-wise comparison provides a more comprehensive evaluation than a single final-point error.

The results show that the FLCM provides comparable accuracy to the FLM at the first comparison point, with relative errors of 0.29% and 0.21%, respectively, at 64,000 cycles. The small difference between the two models is consistent with the limited stiffness degradation during the early fatigue stage. At the subsequent comparison points, the FLCM reduces the relative error from 4.84% to 1.89% at 146,000 cycles and from 7.04% to 2.93% at 214,000 cycles. These results indicate that the advantage of the FLCM becomes more evident as fatigue damage accumulates and structural stiffness degradation becomes more pronounced.

From the perspective of fatigue damage, if no element fails, the structural normalized fatigue stiffness *E*/*E*_0_ will steadily degrade with the fatigue cycle N, such as the stage of ‘fatigue-1’. However, the slope of *E*/*E*_0_ in the green circle 1 (Figure 24b) increases, which corresponds to the failure of compressed elements (Figure 22a). It is obvious that the fatigue cycle of the green circle 1 is very close to the fatigue cycle of the red circle 1, while the fatigue cycle of the blue circle 1 is far away from the fatigue cycle of the red circle 1. This reflects that the fatigue FEM (FLM) underestimates the fatigue life of critical elements, which leads to the premature occurrence of the fiber-compression fatigue failure mode. What’s more, the fatigue FEM (FLM) even experiences a second sudden degradation in the blue circle 2.

The applicability of the proposed FLCM/FSDM-ANN framework is subject to the experimental domain used for its calibration. The present ANN response surfaces were established from T–T and C–C fatigue data for the investigated 3232A/Glass material system and within the corresponding experimentally supported stress–life domain. Therefore, direct extrapolation to other material systems, fiber orientations, loading modes, or substantially different stress states should not be assumed without additional material-level calibration and validation. The structural demonstration presented in this study establishes the applicability of the proposed framework to the investigated flexible composite structure, rather than universal applicability to all flexible composite structures. Further validation using different composite systems, loading modes, and structural configurations is required to assess the broader generality of the method.

## 5. Conclusions and Limitations

A fatigue-life correction model (FLCM) was developed to account for the effect of fatigue stiffness degradation observed in material-level fatigue tests, and a fatigue progressive damage model (FPDM) combining the FLCM with the FSDM-ANN was implemented into the finite element framework. The proposed approach was evaluated using a flexible composite structure (FCS) subjected to multiaxial fatigue loading. The main conclusions are as follows:

(1) The proposed FLCM provides a fatigue-life description that is consistent with the progressive change in material stiffness during cyclic loading. Compared with the traditional FLM, the FLCM provides a more suitable fatigue-life input for the progressive damage analysis of the investigated FCS.
(2) The proposed FPDM reproduced the experimentally observed degradation of structural fatigue stiffness under the investigated three-stage displacement fatigue loading. The normalized structural fatigue stiffness was 0.9530, 0.9047, and 0.8505 at 64,000, 146,000, and 214,000 cycles, respectively. The corresponding maximum prediction error was reduced from 7.04% with the traditional FLM to 2.93% with the proposed FLCM.
(3) The numerical and experimental results indicate that fatigue stiffness degradation of the investigated FCS was mainly associated with progressive damage in the fiber-compression region. The proposed FLCM reduced the premature failure tendency of compressed elements observed when the traditional FLM was used. The predicted failure of compressed elements occurred at approximately 156,000–160,000 cycles, which was close to the experimentally observed change in the structural fatigue-load degradation.
(4) The static and fatigue experiments provided experimental evidence for the structural applicability of the proposed model. In the static tests, the measured ultimate failure loads were 11,630 and 12,094 N, with corresponding displacements of 55.8 and 60.5 mm, respectively. The initial structural stiffness obtained experimentally was 291.72 N/mm, compared with 318.14 N/mm from the FEM. The DIC observations also showed deformation and strain concentration consistent with the corresponding FEM results, with a maximum displacement discrepancy of approximately 4.5%.

Several limitations should be noted. First, only two specimens were used for the static tests and two specimens for the fatigue tests; therefore, the experimental results are intended primarily for model validation rather than statistical characterization of structural fatigue scatter. Second, the fatigue experiment employed a three-stage displacement loading protocol rather than continuous variable-amplitude loading. Third, the present FPDM considers fatigue stiffness degradation but does not explicitly incorporate fatigue strength degradation, which may affect damage initiation and fatigue-life prediction under conditions where residual strength degradation is significant. Fourth, the fatigue tests were conducted at a fixed loading frequency, and the specimen temperature was not monitored during cyclic loading. Therefore, possible temperature variations induced by cyclic loading and their effects on the fatigue response were not explicitly considered in the present validation. Fifth, the FSDM-ANN depends on previously obtained material-level fatigue data and is calibrated for the investigated material system and the corresponding T–T and C–C stress-level domains. Finally, the proposed approach has not yet been independently validated using a different structural geometry or material system.

Future work will therefore focus on independent validation using different composite material systems and structural configurations, extension of the framework to variable-amplitude fatigue loading, investigation of frequency- and temperature-dependent fatigue effects, and development of probabilistic formulations to quantify experimental fatigue scatter and prediction uncertainty.

## Figures and Tables

**Table 1:** Material constants of 3232A/Glass.

Properties (Units)	Value	Properties (Units)	Value
E_1_ (GPa)	68	X_T_ (MPa)	1484
E_2_ (GPa)	25	X_C_ (MPa)	1159
G_12_ (GPa)	4.3	Y_T_ (MPa)	61
S_12_ (MPa)	80	Y_C_ (MPa)	183
ν_12_	0.32		

**Table 2:** Hashin failure criterion and degradation factor.

Failure Mode		Condition	Criterion	Degradation Factor
Fiber damage	tension	*σ*_1_ ≥ 0	${\left(\frac{\sigma_{1}}{X_{T}}\right)}^{2}+{\left(\frac{\tau_{12}}{S_{12}}\right)}^{2}\geq 1$	E_1_*_f_* = 0.1E_1_
	compression	*σ*_1_ ≤ 0	${\left(\frac{\sigma_{1}}{X_{C}}\right)}^{2}\geq 1$	
Matrix damage	crack	*σ*_2_ ≥ 0	${\left(\frac{\sigma_{2}}{Y_{t}}\right)}^{2}+{\left(\frac{\tau_{12}}{S_{12}}\right)}^{2}\geq 1$	E_2_*_f_* = 0.1E_2_ G_12_*_f_* = 0.1G_12_
	compression	*σ*_2_ ≤ 0	${\left(\frac{\sigma_{2}}{Y_{c}}\right)}^{2}+{\left(\frac{\tau_{12}}{S_{12}}\right)}^{2}\geq 1$	
Shear damage	Fiber–Matrix	*σ*_1_ < 0	${\left(\frac{\sigma_{1}}{X_{c}}\right)}^{2}+{\left(\frac{\tau_{12}}{S_{12}}\right)}^{2}\geq 1$	G_12_*_f_* = 0.1G_12_ *ν*_12_*_f_* = 0

Tip: *σ*_1_ and *σ*_2_ represent the stresses in the fiber and transverse directions.

**Table 3:** Characterization of the fitted linear relationships.

Loading	Case	*B*	95% CI	*R* ^2^
T–T	Traditional FLM	0.6390	0.6389–0.6390	>0.9999
T–T	γ = 0.25	0.6921	0.6777–0.7064	0.99995
T–T	γ = 0.50	0.7122	0.6793–0.7451	0.99977
C–C	Traditional FLM	0.7565	0.7565–0.7565	>0.9999
C–C	γ = 0.14	0.8434	0.8348–0.8521	0.99999
C–C	γ = 0.35	0.8798	0.8315–0.9282	0.99967

**Table 4:** Parameters and fatigue-life results used to construct the FLCM under fixed *γ.*

Load	*S*	*γ*	*A*	lg*N_f_*	$\bar{A}$	lg*N_f_* (Equation (10))	lg*N_f_* (Equation (5))	Error *
T–T	75%	0.25	0.4157	3.5502	0.3602	3.6345	3.5494	2.40%
	70%		0.3750	4.2310		4.2533	4.2303	0.54%
	65%		0.3407	4.9836		4.9538	4.9833	0.59%
	60%		0.3094	5.8102		5.7324	5.8113	1.36%
	75%	0.50	0.0844	3.5502	0.0183	3.6503	3.5494	2.84%
	70%		0.0350	4.2304		4.2558	4.2303	0.60%
	65%		−0.0050	4.9832		4.9474	4.9833	0.72%
	60%		−0.0413	5.8116		5.7210	5.8113	1.55%
C–C	85%	0.14	−0.2594	4.5000	−0.3501	4.6186	4.5000	2.64%
	80%		−0.3035	5.0261		5.0871	5.0261	1.21%
	75%		−0.3453	5.5920		5.5981	5.5919	0.11%
	70%		−0.4920	6.1985		6.0130	6.1981	2.99%
	85%	0.35	−1.3164	4.5000	−1.3171	4.5008	4.5000	0.02%
	80%		−1.2694	5.0265		5.0888	5.0261	1.25%
	75%		−1.3150	5.5915		5.5942	5.5919	0.04%
	70%		−1.3674	6.1985		6.1337	6.1981	1.04%

Tip(*): The error is defined as the relative discrepancy in the logarithmic fatigue-life representation: Error = |lg*N_f_*(Equation (10)) – lg*N_f_*(Equation (5))|/lg*N_f_*(Equation (5))*100%.

**Table 5:** Parameters source table.

Quantity	Meaning	Source	Role
*R*	stress ratio	fatigue test	inherited
*S*	initial stress level	fatigue test	inherited
*A*, *B*	FLM fitting constants	traditional FLM	inherited
*γ*	fatigue stiffness degradation parameter	FSDM	inherited/fitted
*N_f_*	fatigue life	experiment/FEM	response
*A_FLCM_*	corrected intercept	bisection	newly determined
*S_eff_*	evolving local stress level	FEM stress state	correction variable

**Table 6:** Configuration and materials of different parts of the FCS.

Global	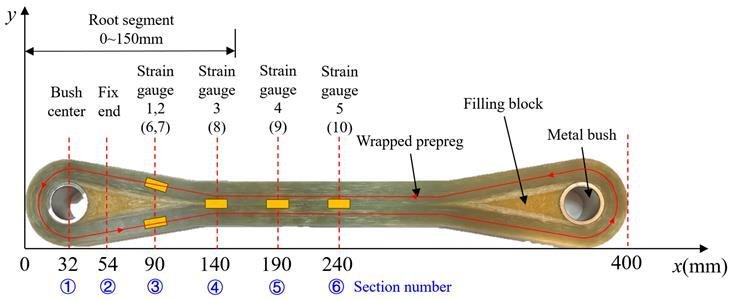		
Parts	Configurations	Materials	Properties
Bush	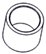	45# steel	E = 213 Gpa *v* = 0.27
Filling block	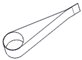	3232A/chopped glass fiber	E = 6.15 Gpa *v* = 0.3
Winding prepreg	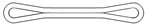	3232A/glass fiber	Table 1, [39]

**Table 7:** Experimental conditions of FCSs.

No.	Load Forms	Frequency	Loading Rate	Flexural Load	Tensile Load	Environment
FCS-S-1	Static	—	2 mm/min	Load to failure	—	RTD
FCS-S-2						
FCS-F-1	Fatigue	0.5 Hz	—	Real-time calibration	5 kN (Const)	
FCS-F-2						

**Table 8:** The three stages of flexural cyclic load of FCS-F-1.

No.	Average Strain in 90 mm Section (με)	Stress Level (%)	Maximum Fatigue Displacement (mm)	Initial Load (N)
1	5104	55.2	26.3	6691
2	5655	61.2	27.5	7470
3	6313	68.3	29.8	8454

**Table 9:** Comparison of the FEM-predicted and experimentally measured responses at the strain-gauge locations.

	90 mm Section	140 mm Section	190 mm Section	240 mm Section
EXPε_11_ (με)	3997	3851	2723	1654
FEM ε_11_ (με)	4298	3995	2629	1577
Error (%)	7.53%	3.74%	3.45%	4.66%

Tips: Error = |(Exp ε_11_ – FEM ε_11_)/Exp ε_11_|*100%.

**Table 10:** Sensitivity of structural fatigue-load degradation to the cycle-jump size.

Δ*N*/Cycles	1000	2000	3000	10,000
Reduction in fatigue load/%	17.11%	17.92%	20.65%	26.42%

**Table 11:** Comparison of normalized structural fatigue stiffness at different fatigue stages.

*N*/Cycles	Experiment (*E*/*E*_0_)	FLM (*E*/*E*_0_)	FLM Error/%	FLCM (*E*/*E*_0_)	FLCM Error/%
64,000	0.9530	0.9511	0.21	0.9503	0.29
146,000	0.9047	0.8610	4.84	0.8876	1.89
214,000	0.8505	0.7906	7.04	0.8256	2.93

## Data Availability

The original contributions presented in this study are included in the article. Further inquiries can be directed to the corresponding author.

## Nomenclature

FLM	Fatigue-Life Model
FLCM	Fatigue-Life Correction Model
FSDM	Fatigue Stiffness Degradation Model
ANN	Artificial Neural Network
FSDM-ANN	Fatigue Stiffness Degradation Model based on Artificial Neural Network
FPDM	Fatigue Progressive Damage Model
FEM	Finite Element Model
3D-FEM	Three-Dimensional Finite Element Model
FCS	Flexible Composite Structure
UMAT	User Material
SDV	Solution-Dependent State Variable
T–T	Tension–Tension
C–C	Compression–Compression
